# An Unusual Rearrangement of Pyrazole Nitrene and Coarctate Ring-Opening/Recyclization Cascade: Formal CH–Acetoxylation and Azide/Amine Conversion without External Oxidants and Reductants

**DOI:** 10.3390/molecules28217335

**Published:** 2023-10-30

**Authors:** Elena Chugunova, Almir S. Gazizov, Daut Islamov, Victoria Matveeva, Alexander Burilov, Nurgali Akylbekov, Alexey Dobrynin, Rakhmetulla Zhapparbergenov, Nurbol Appazov, Beauty K. Chabuka, Kimberley Christopher, Daria I. Tonkoglazova, Igor V. Alabugin

**Affiliations:** 1 Arbuzov Institute of Organic and Physical Chemistry, FRC Kazan Scientific Center, Russian Academy of Sciences, Akad. Arbuzov St. 8, Kazan 420088, Russia; agazizov@iopc.ru (A.S.G.); daut1989@mail.ru (D.I.); vict.matveeva@hotmail.com (V.M.); burilov_2004@mail.ru (A.B.); aldo@iopc.ru (A.D.); ialabugin@gmail.com (I.V.A.); 2 Laboratory of Engineering Profile “Physical and Chemical Methods of Analysis”, Korkyt Ata Kyzylorda University, Aitekebie Str. 29A, Kyzylorda 120014, Kazakhstan; nurgali_089@mail.ru (N.A.); ulagat-91@mail.ru (R.Z.); 3 Zhakhaev Kazakh Scientific Research Institute of Rice Growing, Abay Av. 25B, Kyzylorda 120008, Kazakhstan; 4 Department of Chemistry and Biochemistry, Florida State University, 95 Chieftan Way, Tallahassee, FL 32306-3290, USA; bc21i@fsu.edu (B.K.C.); kmc23@fsu.edu (K.C.); dt22k@fsu.edu (D.I.T.)

**Keywords:** furoxan, 1*H*-pyrazole, nitrene, fragmentation, cyclization

## Abstract

We report an unusual transformation where the transient formation of a nitrene moiety initiates a sequence of steps leading to remote oxidative C–H functionalization (R–CH_3_ to R–CH_2_OC(O)R’) and the concomitant reduction of the nitrene into an amino group. No external oxidants or reductants are needed for this formal molecular comproportionation. Detected and isolated intermediates and computational analysis suggest that the process occurs with pyrazole ring opening and recyclization.

## 1. Introduction

The high energy stored in the azide functionality makes it a powerful tool for many organic transformations [1,2]. In addition, the N_3_ group can be considered a convenient amine surrogate, i.e., a form of amine protection, as this group can be easily installed into a molecule and converted into the corresponding NH_2_ later in the synthetic sequence. Several reductive strategies for azide-to-amine transformations include the use of strong reducing agents, such as LiAlH_4_ [3], NaBH_4_ [4], and Zn(BH_4_)_2_ [5]. The search for milder conditions led to the development of sulfide chemistry [6,7] and copper(I) catalysis [8]. However, the Staudinger reaction with the use of phosphine or phosphite reagents remains the most frequent choice for this reduction [9,10,11,12]. Nevertheless, each of these approaches has its disadvantages, including poor group tolerance, the additional synthesis of ligands, or phosphorus(V) waste.

Although arene azides are most widely presented in the literature, the number of papers dealing with the synthesis and the chemical and biological properties of heterocyclic azides has grown significantly. Now, heterocyclic azides are commonly used as versatile building blocks in organic chemistry [13]. They retain most of the valuable reactivity features typical of their aromatic counterparts as they undergo typical azide transformations, such as the Huisgen cycloaddition [14,15], the CuAAC reaction [16], reduction to amines, 1,3-dipolar cycloaddition to double bonds [17], and so on. However, the neighboring heterocyclic moiety also enables a range of unique transformations. For example, electron-poor imidazolium-based azides may serve as diazo-group transfer reagents [18]. Similar reactivity is observed for super-electrophilic dinitrobenzofuroxan-derived azides [19]. Nenajdenko and coauthors have demonstrated that 4-azido-5-(pyridine-2-yl)-1,2,3-triazoles undergo thermal cyclization to give a novel class of blue-light-emitting heterocycles, 2*H*-[1,2,3]triazolo[4′,5′:3,4]pyrazolo[1,5-*a*]pyridin-5-ium-4-ides [20]. An intriguing example of stable pyrazolo[5,1-*c*][1,2,4]triazine possessing both diazonium and acylazido moieties was reported by Ivanov and coworkers [21]. These selected examples clearly demonstrate the great potential of heterocyclic azides in developing novel transformations.

On the other hand, C–H activation in aliphatic functional groups remains challenging and usually requires oxidative conditions. Furthermore, the direct esterification of C–H bonds is often limited by the nature of the substrates [22,23]. Several oxidative acetoxylation protocols with the in situ generation of hypervalent iodine species have been described [24,25]. The combination of copper [26,27] or palladium catalysts [28,29,30,31,32] with external oxidants or electrochemical oxidation [33] also provided a useful tool for AcO-group insertion into an alkane moiety. However, these approaches are either incompatible with sensitive functional groups (e.g., unprotected amines) or demand the presence of directing groups. Using acetic acid itself for C–H acetoxylation is unusual and conceptually appealing.

**Scheme 1:**
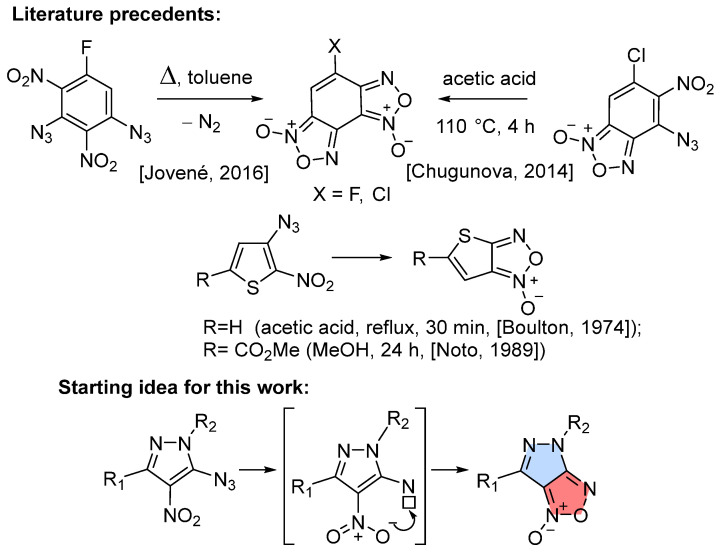
The original goal of this work: fusion of two dinitrogen heterocycles, pyrazole and furoxan, [49,50,51,52].

In continuation of our efforts to search for potent anticancer and antimicrobial agents [34,35,36,37], we were interested in synthesizing a previously unknown bis-heterocyclic pyrazolo-furoxan fusion. Pyrazole-based compounds exert antimicrobial, antipyretic, anti-inflammatory, and analgesic effects [38,39,40,41]. Furoxan-ring-containing molecules exhibit anti-tuberculosis [42], antitumor [43], anti-inflammatory [44], antiaggregant [45], etc., biological activities. For the target compounds’ synthesis, we planned to use one of the known methods for obtaining a furoxan cycle—thermolysis of α-nitroazides (Scheme 1) [46,47,48,49,50,51,52].

However, when we applied these reaction conditions, the outcome was unexpected. The observed transformation involved a remote methyl group and proceeded as a redox disproportionation, where this group was oxidized into a CH_2_OAc moiety while the azido group was reduced to the amine. Interestingly, the nitro group, which could potentially serve as a relay between the two reacting functionalities, remained unchanged. Considering this surprising outcome, we have explored this new conformation in more detail and wish to report the results of this investigation in this work.

## 2. Results and Discussion

**Scheme 2:**
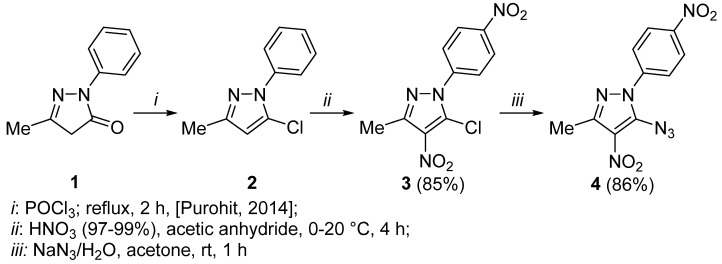
Reaction of edaravone **1** with POCl_3_, following nitration and azidation, with formation of 5-azido-3-methyl-4-nitro-1-(4-nitrophenyl)-1*H*-pyrazole **4 [55]**.

**Synthesis of reactants and analysis of products.** We started by preparing 5-chloro-3-methyl-1-phenyl-1*H*-pyrazole **2** from edaravone **1**, a medication used to treat stroke and amyotrophic lateral sclerosis [53,54] (Scheme 2), according to the literature method [55]. It was reported that the nitration of compound **2** with concentrated nitric acid in acetic anhydride led to nitration at the fourth position of the pyrazole ring with the formation of 5-chloro-3-methyl-4-nitro-1-phenyl-1*H*-pyrazole [55]. When we used fuming nitric acid (97–99%), it resulted in the incorporation of the additional nitro group at the *para*-position of the phenyl group, allowing us to obtain compound **3**.

The reaction of 5-chloro-3-methyl-4-nitro-1-(4-nitrophenyl)-1*H*-pyrazole **3** with sodium azide resulted in the facile replacement of chlorine by the azido group. We have shown earlier that the presence of adjacent azide and nitro groups usually leads to cyclization to the furoxan ring upon refluxing in high-boiling solvents [49,50]. In some cases, this reaction proceeds spontaneously at the stage of azide preparation [56].

**Scheme 3:**
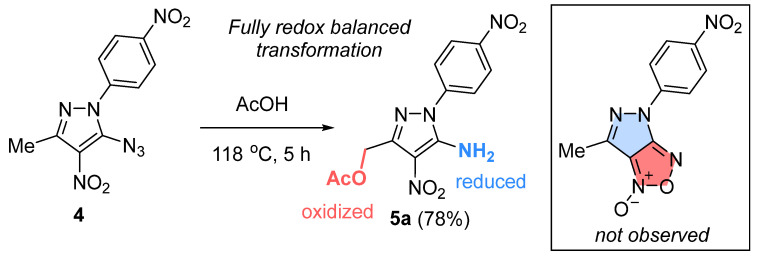
Unexpected redox disproportionation of α-nitroazide **4**.

However, to our surprise, the thermolysis of *o*-azidonitro derivative **4** in acetic acid led to 5-amino-4-nitro-1-(4-nitrophenyl)-1*H*-pyrazol-3-yl)methyl acetate **5a**, instead of the expected cyclization product, furoxan. The new product is formed as a result of the reduction of the azido group to an amino group and the oxidative conversion of the methyl group to a CH_2_OAc moiety (Scheme 3). Aminopyrazole derivatives are widely used in organic synthesis as convenient starting reagents for obtaining new annelated heterocycles, which may be of interest as potentially physiologically active compounds [57]. For example, 4-amino-pyrazole-3-carboxylic esters are used as intermediates for the synthesis of Sildenafil (Viagra) and Allopurinol [58].

**Scheme 4:**
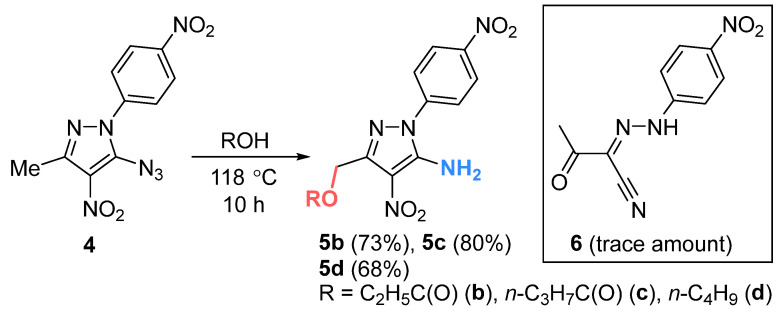
Reaction of nitroazide **4** with selected carboxylic acids and butanol.

The reaction of azide **4** with propionic and butyric acids and butanol leads to similar products **5b**–**d** (Scheme 4). In all cases, compound **6** was isolated as a byproduct in trace amounts.

**Figure 1:**
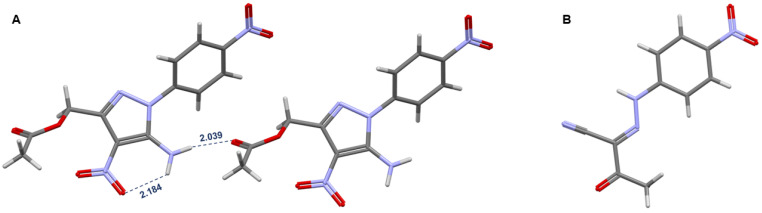
(**A**): Fragment of crystal packing of compound **5a,** (**B**): molecular structure of compound **6**.

The structures of compounds **3**, **5a**, and **6** were confirmed by X-ray analysis (Figures S13, S14 and Figure 1). The bihetaryl scaffold of **5a** is twisted due to steric repulsion in *ortho*-positions (Figure 1A). The interplanar and torsion angles between *p*-nitro-phenylene and pyrazole rings are 37.91° and 36.37°, respectively. The acetoxy group lies in the “*gauche*” conformation to diaza-cycle (torsion angle C_10_C_9_C_12_O_13_ 59.42°), where the acceptor C–O bond aligns with the donor heterocycle π-system. Interestingly, the NH_2_ substituent participates in both intra- and intermolecular hydrogen bonding with oxygens of NO_2_ and C=O groups, respectively.

Hydrazide **6** has a completely planar structure with two cross-conjugated fragments at the NH moiety (Figure 1B). A lone electron pair of the NH nitrogen can participate in conjugation with both substituents, and as a result, the central N-atom is sp^2^-hybridized. The sum of valence angles at the NH nitrogen equals 360.0°.

**Scheme 5:**
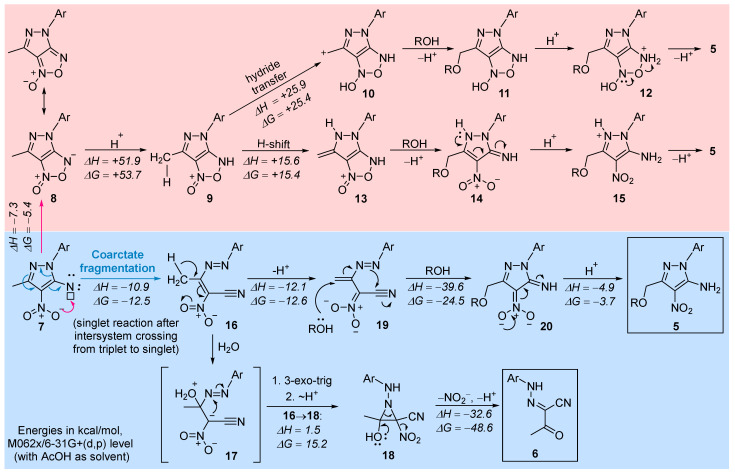
Suggested mechanism for the formation of unusual products in the attempted synthesis of pyrazole/furoxan hybrid. Pathways discarded from the computational evidence are shown in the red background. The suggested plausible path is shown in blue. Both cyclization and fragmentation proceed from the singlet state.

**Possible mechanism.** Considering that this process involves a metal-free C–H activation under relatively mild conditions, its mechanism is intrinsically interesting, as it may pave the way to similar C–H activations of a broader range of substrates. The key step is likely to be the formation of the nitro-substituted nitrene (Scheme 5). Aryl nitrenes are capable of complex transformations, including a variety of ring expansions, fragmentations, and bond insertions [59,60,61,62]. Singlet aryl nitrenes can also be trapped by reactions with internal nucleophiles [63,64,65]. Although Ph-nitrene is known to be a triplet (~15 kcal/mol lower than the open-shell singlet state) [66], the singlet nitrene was shown to be a discrete, albeit very short-lived, intermediate [67], capable of fast intramolecular reactions. On the other hand, the singlet/triplet gap in pyrazole nitrene has not been studied. Our attempts to optimize the singlet state of pyrazole nitrene lead to its barrierless fragmentation via a coarctate reaction (vide infra).

Once the pyrazole nitrene **7** is formed, it can potentially react in several ways. The direct intramolecular attack of an oxygen of the nitro group leads to neutral bicyclic species **8**, which can be converted into **9** by protonation. Alternatively, pyrrole nitrogen can increase the electron density at the nitrene via resonance, facilitating protonation with the formation of a nitrenium ion (not shown), which may also cyclize with the formation of the fused bicyclic cation **9**. When **9** is formed, the mechanistic path can diverge. Here, we have considered two possibilities. First, the intramolecular activation of the methyl substituent by electron-deficient oxygen in **9** may enable hydride transfer, allowing for the formation of a stabilized carbocation **10**. The nucleophilic attack of ROH at the cationic carbon, followed by proton transfer and furoxan ring opening, would form the final product **5**. An interesting feature of this path would be that nitrene would provide the transient activation of the nitro group, rendering it an even stronger hydride acceptor. However, computational analysis at the M062x/6-31+G (d,p) level (with AcOH as a solvent) suggested that the barrier to such a hydride shift is prohibitively large (67 kcal/mol), and the carbocationic intermediate **10** is nearly 25 kcal/mol higher in energy than **9**.

In an alternative intramolecular activation path, the prototropic tautomerization of cation **9** in an acidic medium would lead to the dearomatizing methyl–methylene tautomeric transformation **9**→**13**. ROH attack on the alkene fragment can synchronously open the furoxan ring and unmask the nitro and imine groups of **14**. Subsequent proton transfers reestablish aromaticity with the formation of pyrazole **15**. The deprotonation of **15** would lead to the observed product **5**. We consider this path unfavorable due to the >15 kcal/mol penalty for the loss of aromaticity in **13**. Furthermore, the formation of the key precursor, i.e., the bicyclic cation **9**, by the protonation of neutral **8** by acetic acid is also predicted to be >50 kcal/mol uphill.

Based on these considerations and the formation of the acyclic side product **6**, we explored the possibility of ring opening as the first step in the reaction sequence. Here, we were guided by the literature precedent, as 5-azidopyrazoles are known to undergo ring-opening processes with the formation of a cyano group after nitrogen loss upon heating [68,69]. Furthermore, our attempts to optimize the singlet state of nitrene **7** led to its barrierless fragmentation with the formation of acyclic nitrile **16**.

The formation of the byproduct **6** can start as a Michael addition of water to an alkene **16** strongly activated by two acceptor groups (nitro and cyano). The adduct **17** can undergo a 3-exo-trig cyclization to afford N-amino-substituted aziridine **18**. Due to hybridization effects [70] and an inverse α-effect [71,72,73], such species are expected to be quite strained and reactive. The collapse of amino acetal with the concomitant aziridine ring opening in **18** relieves the transient strain. The hydrazine lone pair then assists in the elimination of the NO_2_ group to reestablish conjugation and, after deprotonation, forms the isolated side product **6**.

Furthermore, the same intermediate **16** can undergo isomerization into alkene **19**, which, after the nucleophilic addition of ROH, can undergo a 5-exo-trig ring closure that recreates the heterocyclic moiety. The protonation of the cyclic nitronate **20** is coupled with aromatization and the formation of the final product **5**. Note that the difference between the two blue pathways is that, in one of them, water serves as a base (formation of **5**), and in the other one, water is a nucleophile (generation of **6**). Hence, the diverging paths leading to the cyclic product and acyclic side product are logically connected.

**Scheme 6:**
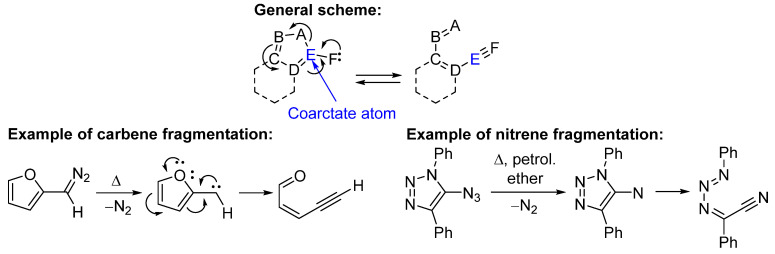
General coarctate ring-opening reaction induced by an electron-deficient and coordinatively unsaturated atom, e.g., a carbene or a nitrene. The parent carbene/nitrene species can be formed via decomposition of diazo compounds or azides, respectively.

The ring-opening/ring-closure mechanism of five-membered rings is an example of a coarctate reaction introduced in the pioneering work by Herges [74]. In these reactions, a coarctate atom forms two bonds and breaks two bonds simultaneously in a concerted fashion (Scheme 6). The presence of an exocyclic electron-deficient atom (nitrene or carbene) results in ring fragmentation with ene-ene-yne moiety formation [75]. Reactions of carbenes generally lead to alkynes, while nitrenes generate nitriles. Unlike alkynes [76], nitriles are a low-energy functionality (an “energy sink”), so nitrene fragmentations are generally quite favorable [75].

The coarctate ring opening provides an interesting counterpart to the ANRORC mechanism [77]. Although it is mechanistically different from ANRORC, it also illustrates that even stable aromatic/heteroaromatic rings are not immune under conditions where highly reactive intermediates are formed, especially when there is a direct path to a stable functional group, such as the CN moiety. Ring openings often lead to recyclizations into new heterocyclic structures [78]. The unusual feature of our work is that the recyclization is accompanied by selective C–H functionalization while retaining the parent heteroaromatic moiety.

In conclusion, we report an interesting “redox-balanced” transformation where two functional groups separated in space undergo simultaneous redox transformations in opposite directions: the Me group is oxidized while the nitrene moiety is reduced. The absence of usual furoxan products in this case can be attributed to the combination of two factors: the lower aromaticity of pyrazole relative to benzene [79,80] and the accumulation of strain upon the fusion of the two five-membered rings [81]. The interplay of electronic effects due to the presence of multiple nitrogen atoms in pyrazole activates the fast Grob fragmentation into a functionally rich acyclic nitro nitrile 16, which can recyclize after prototropic isomerization and the Michael-like addition of AcOH.

## 3. Materials and Methods

### Chemistry

IR spectra were recorded as an emulsion in vaseline oil (sample concentration 0.25%) on a Tensor 37 Vertex 70 RAM II spectrometer (Bruker Optik GmbH, Ettlingen, Germany) in the range 400–4000 cm^−1^; given are the most intense absorption bands. Nuclear magnetic resonance (NMR) spectra were recorded on a Bruker AVANCE 400 spectrometer (Bruker BioSpin, Rheinstetten, Germany) operating at 400 MHz (for ^1^H NMR) and 101 MHz (for ^13^C NMR) and on a Brucker AVANCE*III*-500 spectrometer (Bruker BioSpin, Rheinstetten, Germany) operating at 500.1 MHz for ^1^H at 303 K and 126 MHz (for ^13^C NMR). Chemical shifts were measured in δ (ppm) with reference to the solvent (δ = 7.27 ppm and 77.00 ppm for CDCl_3_, δ = 2.06 ppm and 28.94 ppm for (CD_3_)_2_CO for ^1^H and ^13^C NMR, respectively). Electrospray ionization (ESI) mass spectra were obtained on an Amazon X mass spectrometer from Bruker Daltonics (Bremen, Germany) with an ion trap. The measurements were carried out in the mode of recording negative ions in the *m*/*z* range from 100 to 2000. Elemental analysis was performed on a CHNS-O Elemental Analyzer EuroEA3028-HT-OM (EuroVector S.p.A., Milan, Italy) with an accuracy of ±0.4% for C, H, Cl, and N. The melting point was determined in glass capillaries on a Stuart SMP 10 instrument (Keison Products, Chelmsford, UK). The progress of reactions and the purity of products were monitored by TLC on Sorbfil UV-254 plates (Sorbpolimer, Krasnodar, Russia). The visualization of the TLC plates was accomplished with a UV light. All standard reagents were purchased from Aldrich or Acros Organics and used without further purification. 5-Chloro-3-methyl-1-phenyl-1*H*-pyrazole **2** was obtained according to a previously described procedure [55].

X-ray crystallography data. The data set for the single crystals **3** and **6** were collected on a Bruker Quest diffractometer using graphite monochromated MoKα (0.71073 Å) radiation and ω-scan rotation. Data collection: images were indexed, integrated, and scaled using the APEX2 [82] data reduction package and corrected for absorption using SADABS [83]. Structure **3** was solved by direct methods and refined using SHELX [84]. Non-hydrogen atoms were refined anisotropically. Hydrogen atoms were calculated in idealized positions and refined as riding atoms. The X-ray analysis was performed on the equipment of the Spectral-Analytical Center of FRC Kazan Scientific Center of RAS.

Crystallographic data for compound **3**: C_10_H_7_ClN_4_O_4_, *M* 282.65, monoclinic, *P*2_1_/*c*, *a* 3.7604(1), *b* 32.2193(11), *c* 9.0673(3) Å, β 93.985(1)°, *V* 1095.92(6) Å^3^, *Z* 4, *D*_calcd_ 1.713 g·cm^–3^, *μ*(Mo-*K*α) 0.367 mm^–1^, F(000) 576, (θ 1.3–27.9°, completeness 99.9%), T 100(2) K, orange prism, (0.11 × 0.17 × 0.56) mm^3^, transmission 0.6946–0.7456, 39,950 measured reflections, 2597 independent (*R*_int_ 0.044), 173 parameters, *R*_1_ = 0.0367 (for 2379 observed *I >* 2*σ*(*I*)), *wR*_2_ = 0.1435 (all data), GOOF 1.05, largest diff. peak and hole 0.50 and −0.40 e·A^−3^. CCDC number 2299127.

Crystallographic data for compound **6**: C_10_H_8_N_4_O_3_, *M* 232.20, monoclinic, *P*2_1_*/n*, *a* 7.0578(14), *b* 13.434(3), *c* 11.160(2) Å, β 96.736(6)°, *V* 1050.8(4) Å^3^, *Z* 4, *D*_calcd_ 1.468 g·cm^–3^, *μ*(Mo-*K*α) 0.113 mm^–1^, F(000) 480, (θ 2.4–27.9°, completeness 99.8%), T 162(2) K, orange needle, (0.04 × 0.05 × 0.15) mm^3^, transmission 0 0.6291–0.7456, 39,950 measured reflections, 23,472 independent (*R*_int_ 0.273), 155 parameters, *R*_1_ = 0.0852 (for 1013 observed *I >* 2*σ*(*I*)), *wR*_2_ = 0.2504 (all data), GOOF 0.941, largest diff. peak and hole 0.31 and −0.28 e·A^−3^. CCDC number 2299128.

The data set for the single crystal **5a** was collected on a Rigaku Synergy S instrument (Rigaku Oxford diffraction, Tokyo, Japan) with a HyPix detector and a PhotonJet microfocus X-ray tube using Cu Kα (1.54184 Å) radiation at a low temperature. Images were indexed and integrated using the CrysAlisPro data reduction package. Data were corrected for systematic errors and absorption using the ABSPACK module: numerical absorption correction based on Gaussian integration over a multifaceted crystal model and empirical absorption correction based on spherical harmonics according to point-group symmetry using equivalent reflections. The GRAL module was used for the analysis of systematic absences and space-group determination. The structure was solved by direct methods using SHELXT [85] and refined by full-matrix least-squares on F2 using SHELXL [86]. Non-hydrogen atoms were refined anisotropically. The hydrogen atoms were inserted at the calculated positions and refined as riding atoms. The figures were generated using the Mercury v4.1 [87] program. Crystals were obtained by the slow evaporation method.

Crystallographic data for compound **5a**: C_12_H_11_N_5_O_6_ (*M* = 321.26 g/mol): triclinic, space group P-1 (no. 2), *a* = 7.7774(2) Å, *b* = 9.7999(2) Å, *c* = 9.96110(10) Å, *α* = 102.833(2)°, β = 111.543(2)°, γ = 92.620(2)°, *V* = 681.72(3) Å^3^, *Z* = 2, *T* = 110.0(5) K, μ(Cu Kα) = 1.107 mm^−1^, *Dcalc* = 1.565 g·cm^−3^, 7375 reflections measured (9.348° ≤ 2Θ ≤ 153.212°), 2745 unique (*R*_int_ = 0.0229, R_sigma_ = 0.0227), which were used in all calculations. The final *R*_1_ was 0.0347 (I > 2σ(I)), and *wR*_2_ was 0.0938 (all data). CCDC number 2294752.

CCDC 2299127, 2299128, and 2294752 contain the supplementary crystallographic data for this paper. These data can be obtained free of charge via https://www.ccdc.cam.ac.uk/structures/ (accessed 20 October 2023) (or from the Cambridge Crystallographic Data Centre, 12 Union Road, Cambridge CB2 1EZ, UK; fax: (+44) 1223-336-033; or deposit@ccdc.cam.uk).

*5-Chloro-3-methyl-4-nitro-1-(4-nitrophenyl)-1H-pyrazole* (**3**)**.** Acetic anhydride (6 mL) was added to 5-chloro-3-methyl-1-phenyl-1*H*-pyrazole **2** (0.44 g, 2.3 mmol), the reaction mixture was cooled to 0 °C, and then fuming nitric acid (97–99%, 4 mL) was added dropwise. The reaction mixture was stirred at room temperature for 4 h and then poured over crushed ice. The obtained precipitate was filtered off, washed with cold water (100 mL), and dried under vacuum (0.06 mm Hg) at 40 °C to constant weight. The crude product was recrystallized from acetone to give the target compound. Yellow powder, yield 0.55 g (85%), m.p.: 148–150 °C. IR (ν, cm^–1^): 691, 787, 860, 1005, 1147, 1317, 1346 (NO_2_ symm), 1381, 1459, 1503, 1532 (NO_2_ asymm), 1553, 1596. ^1^H NMR (500 MHz, CDCl_3_): δ = 8.41 (d, *J* = 8.6 Hz, 2H), 7.83 (d, *J* = 8.6 Hz, 2H), 2.64 (s, 3H). ^13^C NMR (126 MHz, CDCl_3_): δ = 148.7, 147.9, 141.7, 131.2, 128.2, 126.0, 125.0, 14.7. Anal. calcd (%) for C_10_H_7_ClN_4_O_4_: C, 42.50; H, 2.50; Cl, 12.54; N, 19.82. Found: C, 42.54; H, 2.48; Cl, 12.53; N, 19.85.
*5-Azido-3-methyl-4-nitro-1-(4-nitrophenyl)-1H-pyrazole* (**4**). To a solution of 5-chloro-3-methyl-4-nitro-1-(4-nitrophenyl)-1*H*-pyrazole **3** (0.50 g, 1.8 mmol) in acetone (5 mL) at room temperature was added a solution of sodium azide (0.15 g, 2.3 mmol) in 1 mL of water. The reaction mixture was stirred for 1 h (the reaction was monitored by thin-layer chromatography; eluent: toluene–ethylacetate (2:1, *v*/*v*)). After completion of the reaction, the solvent was removed under reduced pressure, washed with cold water, and dried in vacuum (0.06 mm Hg) at 40 °C to constant weight. Light brown powder, yield 0.45 g (86%), R_f_ 0.31, m.p.: 104–106 °C. IR (ν, cm^–1^): 690, 751, 822, 857, 1347 (NO_2_ symm), 1382, 1418, 1439, 1557 (NO_2_ asymm), 1561, 2152 (N_3_). ^1^H NMR (500 MHz, Acetone-*d*_6_): δ = 8.40–8.43 (m, 2H), 8.07–8.11 (m, 2H), 2.55 (s, 3H). ^13^C NMR (101 MHz, Acetone-*d*_6_): δ = 147.9, 147.6, 142.5, 138.5, 126.2, 125.2, 125.1, 14.4. Anal. calcd (%) for C_10_H_7_N_7_O_4_: C, 41.53; H, 2.44; N, 33.90. Found: C, 41.58; H, 2.47; N, 33.87.

**Synthesis of compounds 5a–d** (**general method**). 5-Azido-3-methyl-4-nitro-1-(4-nitrophenyl)-1*H*-pyrazole **4** (0.1 g, 0.34 mmol) was heated in 3 mL of acid/butanol at 118 °C for 5 h (for acetic acid) or 10 h (for propionic and butyric acids and butanol). Then, the solvent was removed under reduced pressure. In the case of propionic and butyric acids and butanol, the crude product was purified by column chromatography on silica gel (eluent: toluene–ethylacetate (10:1, *v*/*v*)) to give the target compound (the side product **6** was isolated in trace amounts).

*5-Amino-4-nitro-1-(4-nitrophenyl)-1H-pyrazol-3-yl)methyl acetate* (**5a**)**.** Gray pearlescent solid (0.08 g) was obtained in 78% yield. R_f_ 0.17, m.p.: 198–199 °C. IR (ν, cm^–1^): 820, 863, 1032, 1253, 1346 (NO_2_ symm), 1464, 1520, 1599 (NO_2_ asymm), 1637 (CO), 1721 (C=O), 3293, 3408 (NH_2_). ^1^H NMR (400 MHz, Acetone-*d*_6_): δ = 8.46 (d, *J* = 9.0 Hz, 2H), 7.99 (d, *J* = 9.0 Hz, 2H), 7.35 (br.s, 2H), 5.33 (s, 2H), 2.08 (s, 3H). ^13^C NMR (101 MHz, Acetone-*d*_6_): δ = 169.6, 147.1, 144.7, 142.3, 125.1, 124.9, 116.9, 58.7, 19.6. Anal. calcd (%) for C_12_H_11_N_5_O_6_: C, 44.87; H, 3.45; N, 21.80. Found: C, 44.83; H, 3.52; N, 21.85. ESI, *m*/*z* for C_12_H_11_N_5_O_6_: 319.99 [M − H]^−^.
*(5-Amino-4-nitro-1-(4-nitrophenyl)-1H-pyrazol-3-yl)methyl propionate* (**5b**)**.** Orange oil, yield 0.085 g (73%). R_f_ 0.15. IR (ν, cm^–1^): 694, 753, 819, 860, 1099, 1182, 1291, 1347 (NO_2_ symm), 1460, 1526, 1598 (NO_2_ asymm), 1635 (CO), 1708, 1738 (C=O), 3328, 3430 (NH_2_). ^1^H NMR (400 MHz, Acetone-*d*_6_): δ = 8.38–8.42 (m, 2H), 7.91–7.97 (m, 2H), 7.36 (br.s, 2H), 5.31 (s, 2H), 2.38 (q, *J* = 7.6 Hz, 2H), 1.11 (t, *J* = 7.6 Hz, 3H). ^13^C NMR (101 MHz, Acetone-*d*_6_): δ = 173.9, 147.9, 145.7, 143.1, 125.9(4), 125.9(1), 125.6, 117.7, 59.5, 27.7, 9.4. Anal. calcd (%) for C_13_H_13_N_5_O_6_: C, 46.57; H, 3.91; N, 20.89. Found: C, 46.72; H, 4.02; N, 20.92. ESI, *m*/*z* for C_13_H_13_N_5_O_6_: 334.04 [M − H]^−^.
*(5-Amino-4-nitro-1-(4-nitrophenyl)-1H-pyrazol-3-yl)methyl butyrate* (**5c**). Orange oil, yield 0.096 g (80%). R_f_ 0.27. IR (ν, cm^–1^): 753, 769, 821, 860, 1012, 1110, 1178, 1290, 1347 (NO_2_ symm), 1459, 1526, 1598 (NO_2_ asymm), 1634 (CO), 1705, 1734 (C=O), 3324, 3434 (NH_2_). ^1^H NMR (600 MHz, Acetone-*d*_6_): δ = 8.42–8.48 (m, 2H), 7.95–8.01 (m, 2H), 7.36 (br.s, 2H), 5.33 (s, 2H), 2.35 (t, *J* = 7.4 Hz, 2H), 1.65 (q, *J* = 7.4 Hz, 2H), 0.95 (t, *J* = 7.4 Hz, 3H). ^13^C NMR (126 MHz, Acetone-*d*_6_): δ = 173.1, 148.0, 147.9, 145.7, 143.2, 125.9(5), 125.8(9), 125.7, 59.4, 36.3, 19.1, 13.8. Anal. calcd (%) for C_14_H_15_N_5_O_6_: C, 48.14; H, 4.33; N, 20.05. Found: C, 48.20; H, 4.37; N, 20.01. ESI, *m*/*z* for C_14_H_15_N_5_O_6_: 348.05 [M − H]^−^.
*3-(Butoxymethyl)-4-nitro-1-(4-nitrophenyl)-1H-pyrazol-5-amine* (**5d**)**.** Orange oil, yield 0.075 g (68%). R_f_ 0.30. IR (ν, cm^–1^): 694, 753, 770, 820, 860, 1013, 1111, 1172, 1290, 1346 (NO_2_ symm), 1457, 1525, 1598 (NO_2_ asymm), 1634 (CO), 1702 (C=O), 3330, 3430 (NH_2_). ^1^H NMR (400 MHz, Acetone-*d*_6_): δ = 8.41–8.45 (m, 2H), 7.94–7.99 (m, 2H), 7.29 (br.s, 2H), 4.68 (s, 2H), 3.59 (t, *J* = 6.5 Hz, 2H), 1.62–1.53 (m, 2H), 1.48–1.32 (m, 2H), 0.89 (t, *J* = 7.4 Hz, 3H). ^13^C NMR (101 MHz, Acetone-*d*_6_): δ = 147.9, 147.7(4), 147.6(8), 143.4, 125.9, 125.6, 117.9, 71.3, 65.9, 32.5, 19.9, 14.1. Anal. calcd (%) for C_14_H_17_N_5_O_5_: C, 50.15; H, 5.11; N, 20.89. Found: C, 50.23; H, 5.17; N, 20.82. ESI,) *m*/*z* for C_14_H_17_N_5_O_5_: 334.08 [M − H]^−^.

## Data Availability

Data is contained within the article and supplementary Materials.

## Supplementary Materials

The following supporting information can be downloaded at https://www.mdpi.com/article/10.3390/molecules28217335/s1, Figures S1–S12 (p. 2–7)—copies of NMR spectra of all synthesized compounds; S13–S14 (p. 8)—the X-ray diffraction data of compound **3**; p. 9–24—computational data; p. 25—references for computational data. Refs. [88,89,90,91,92] are cited in Supplementary Materials.
