# Supplementary material for: An Unusual Rearrangement of Pyrazole Nitrene and Coarctate Ring-Opening/Recyclization Cascade: Formal CH–Acetoxylation and Azide/Amine Conversion without External Oxidants and Reductants
Source: Molecules. 2023 Oct 30;28(21):7335. doi: 10.3390/molecules28217335 (PMC10648078; doi:10.3390/molecules28217335)
Supplement: Supplementary file 1 [file molecules-28-07335-s001.zip › molecules-2677914-supplementary.pdf]

Supplementary Material

An Unusual Rearrangement of Pyrazole Nitrene and Coarctate Ring-Opening/Recyclization Cascade: Formal CH–Acetoxylation and Azide/Amine Conversion without External Oxidants and Reductants

Elena Chugunova [1,*], Almir S. Gazizov [1], Daut Islamov [1], Victoria Matveeva [1], Alexander Burilov [1], Nurgali Akylbekov [2], Alexey Dobrynin [1], Rakhmetulla Zhapparbergenov [2], Nurbol Appazov [2,3,*], Beauty K. Chabuka [4], Kimberley Christopher [4], Daria I. Tonkoglazova [4] and Igor V. Alabugin [1,4]

[1] Arbuzov Institute of Organic and Physical Chemistry, FRC Kazan Scientific Center, Russian Academy of Sciences, Akad. Arbuzov St. 8, Kazan 420088, Russia; agazizov@iopc.ru (A.S.G.); daut1989@mail.ru (D.I.); vict.matveeva@hotmail.com (V.M.); burilov_2004@mail.ru (A.B.); aldo@iopc.ru (A.D.); ialabugin@gmail.com (I.V.A.)
[2] Laboratory of Engineering Profile "Physical and Chemical Methods of Analysis", Korkyt Ata Kyzylorda University, Aitekebie Str. 29A, Kyzylorda 120014, Kazakhstan; nurgali_089@mail.ru (N.A.); ulagat-91@mail.ru (R.Z.)
[3] Zhakhaev Kazakh Scientific Research Institute of Rice Growing, Abay Av. 25B, Kyzylorda 120008, Kazakhstan
[4] Department of Chemistry and Biochemistry, Florida State University, 95 Chieftan Way, Tallahassee, FL 32306-3290, USA; bc21i@fsu.edu (B.K.C.); kmc23@fsu.edu (K.C.); dt22k@fsu.edu (D.I.T.)
* Correspondence: chugunova.e.a@gmail.com (E.C.); nurasar.82@mail.ru (N.A.); Tel.: +7-843-272-7324 (E.C.); +8(7242)-23-10-41 (N.A.)

Experimental details, copies of NMR spectra, X-ray crystallographic data, computational details

Table of contents

Copies of ^{1}H and ^{13}C NMR spectra (Figs. S1-S12) 2
X-ray data (Figs. S13-S14) 8
Computational details 9
References 25

NMR spectra of compounds

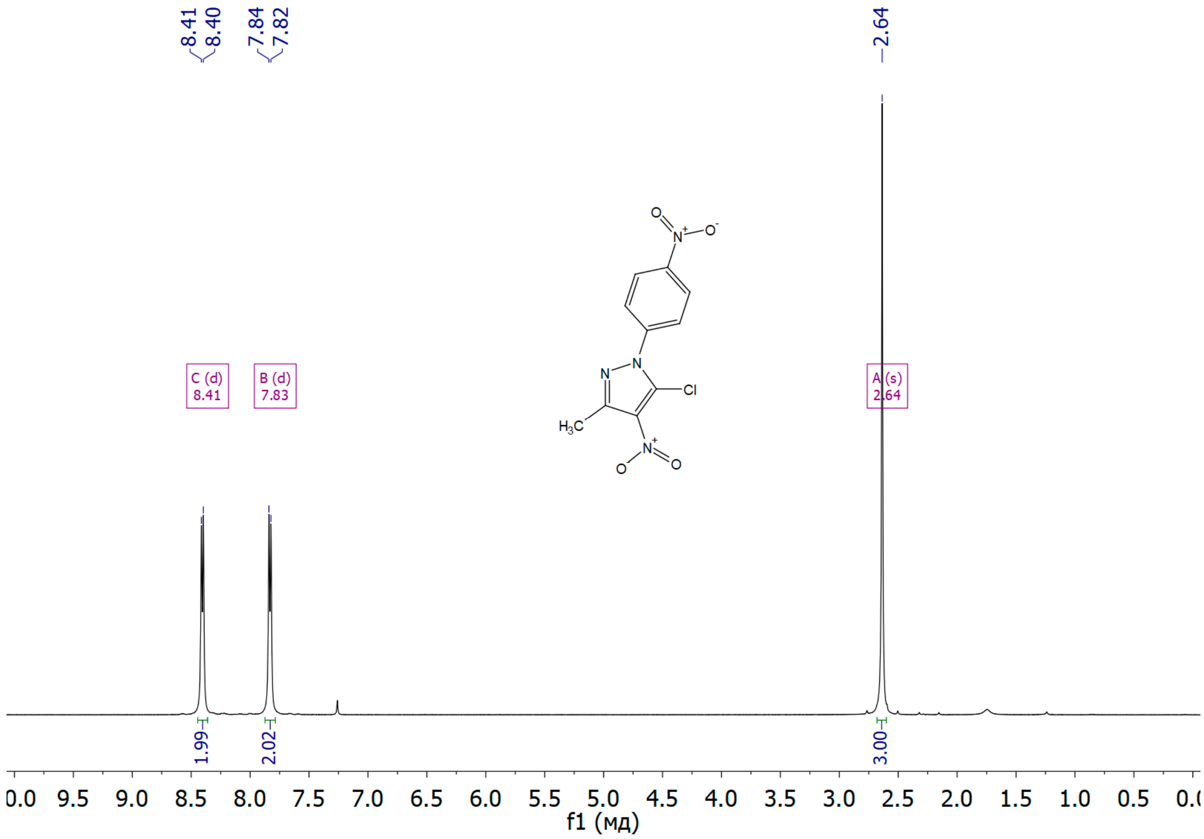

Figure S1.^{1}H NMR spectrum of **3** ($CDCl_3$, 500 MHz)

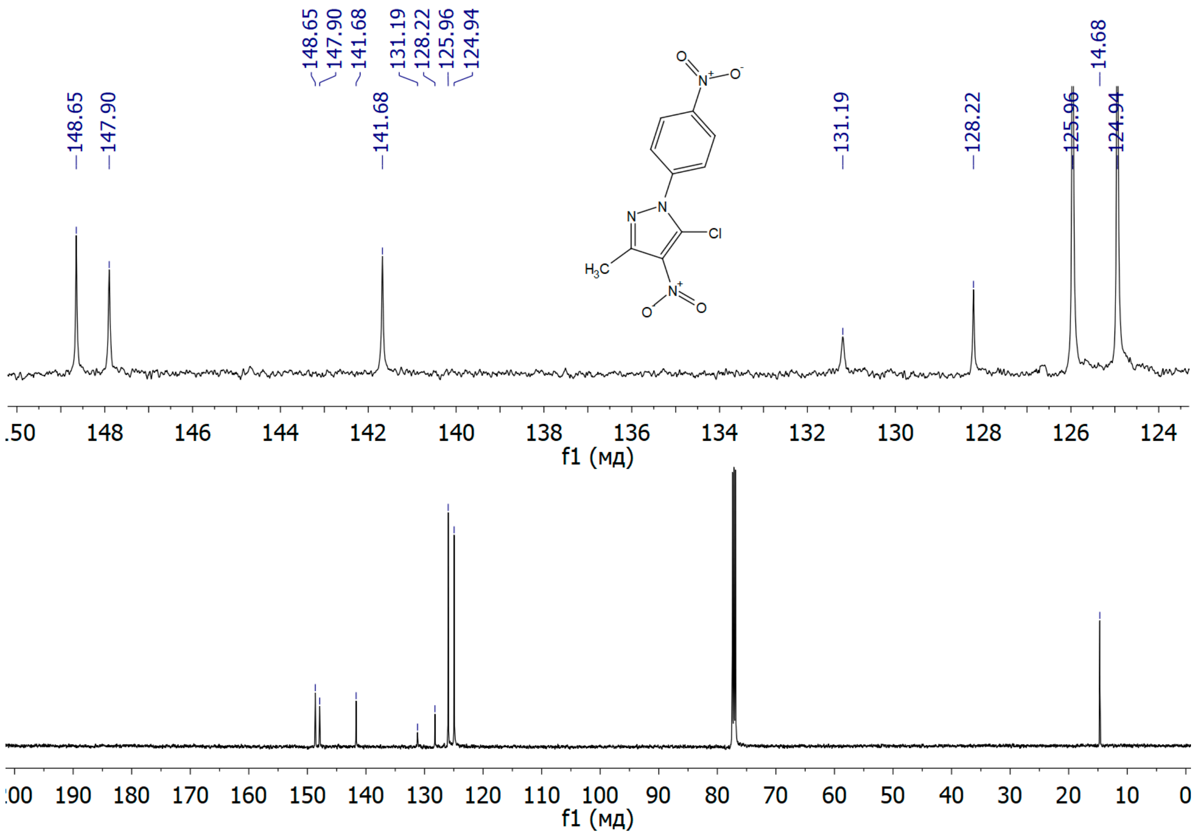

Figure S2.^{13}C NMR spectrum of **3** ($CDCl_3$, 126 MHz)

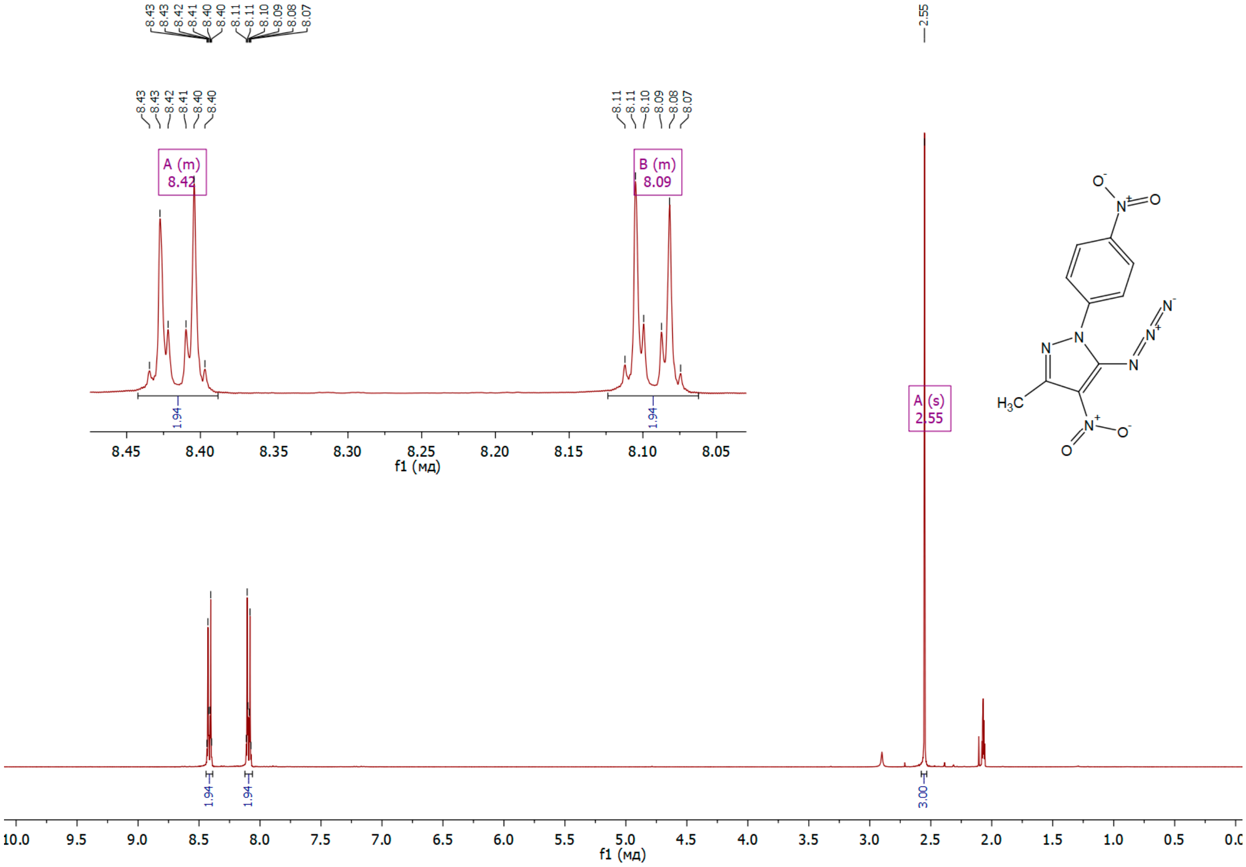

Figure S3.^{1}H NMR spectrum of **4** (Acetone-d_6, 500 MHz)

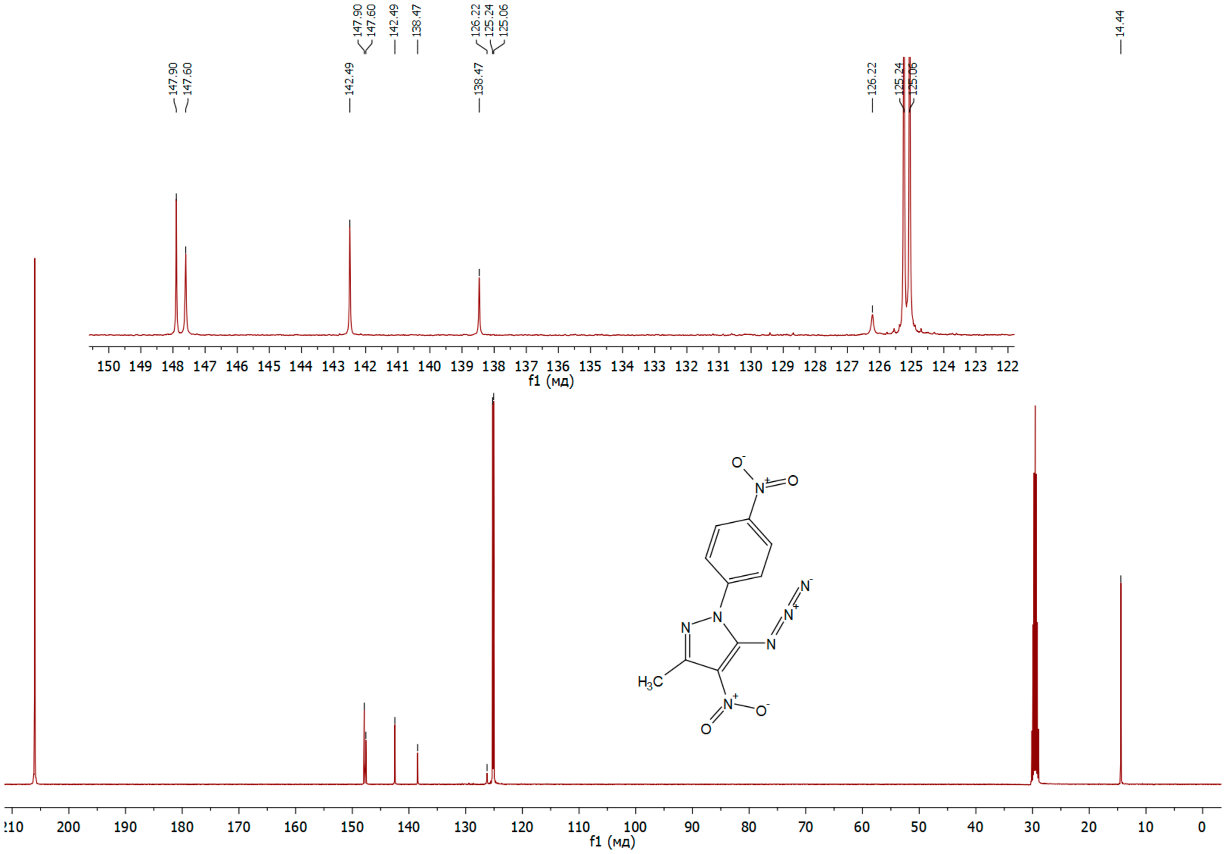

Figure S4.^{13}C NMR spectrum of **4** (Acetone-d_6, 101 MHz)

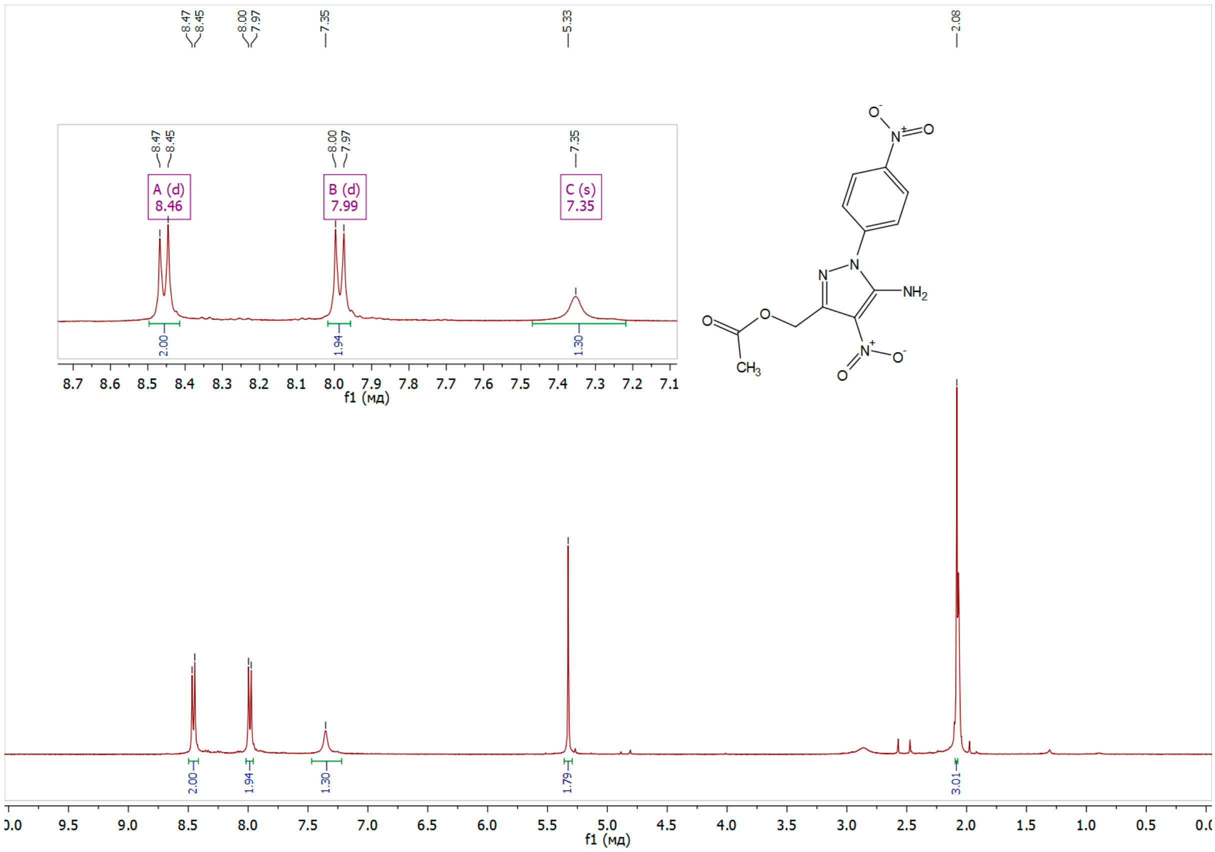

Figure S5. ^{1}H NMR spectrum of **5a** (Acetone-d_6, 400 MHz)

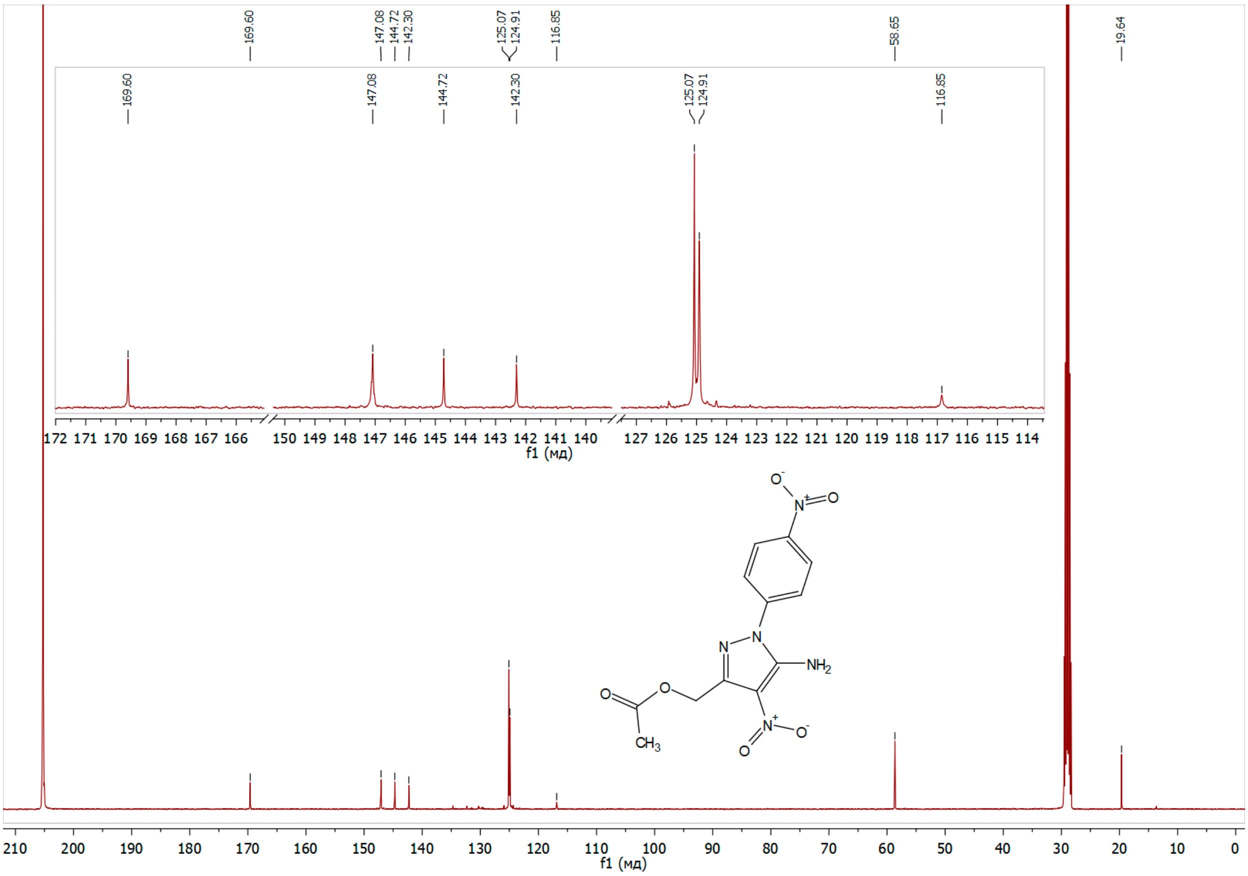

Figure S6. ^{13}C NMR spectrum of **5a** (Acetone-d_6, 101 MHz)

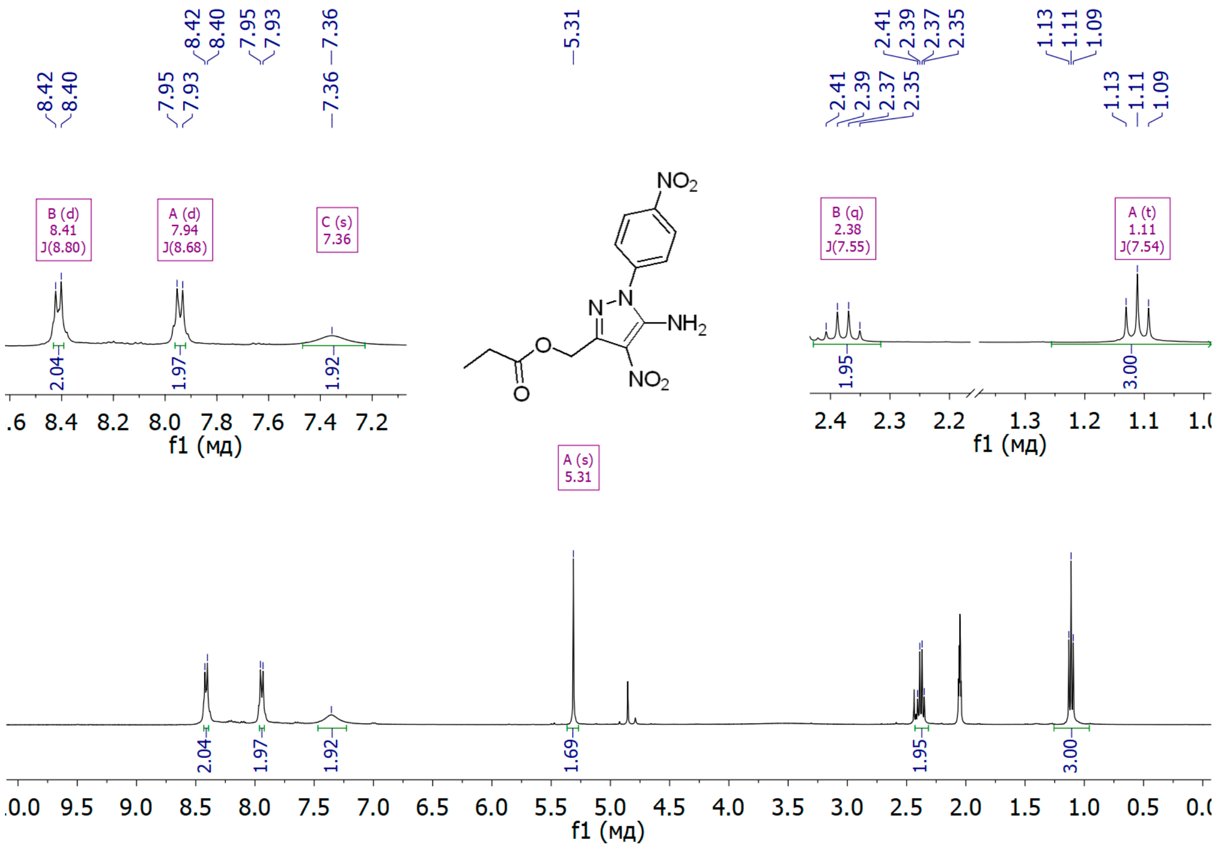

Figure S7.^{1}H NMR spectrum of **5b** (Acetone-d_6, 400 MHz)

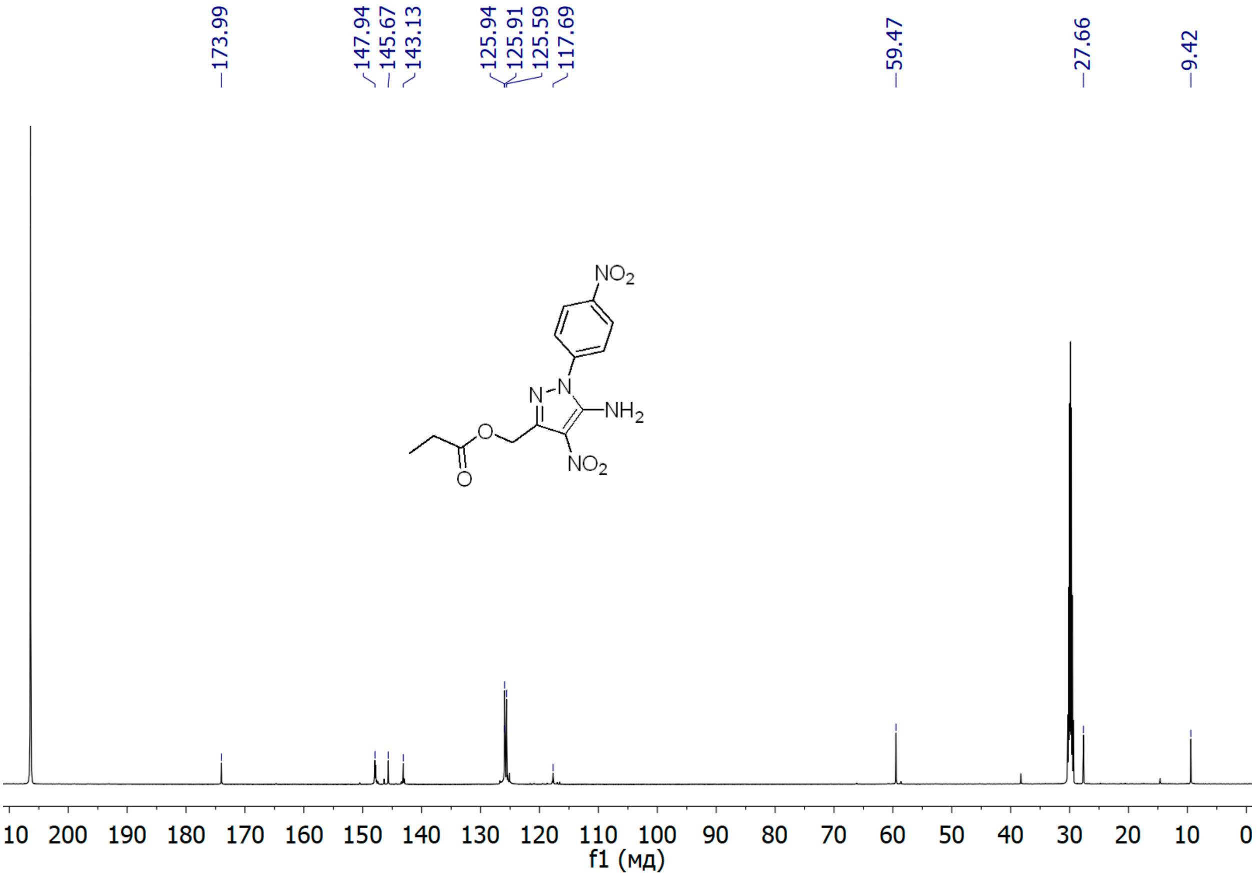

Figure S8.^{13}C NMR spectrum of **5b** (Acetone-d_6, 101 MHz)

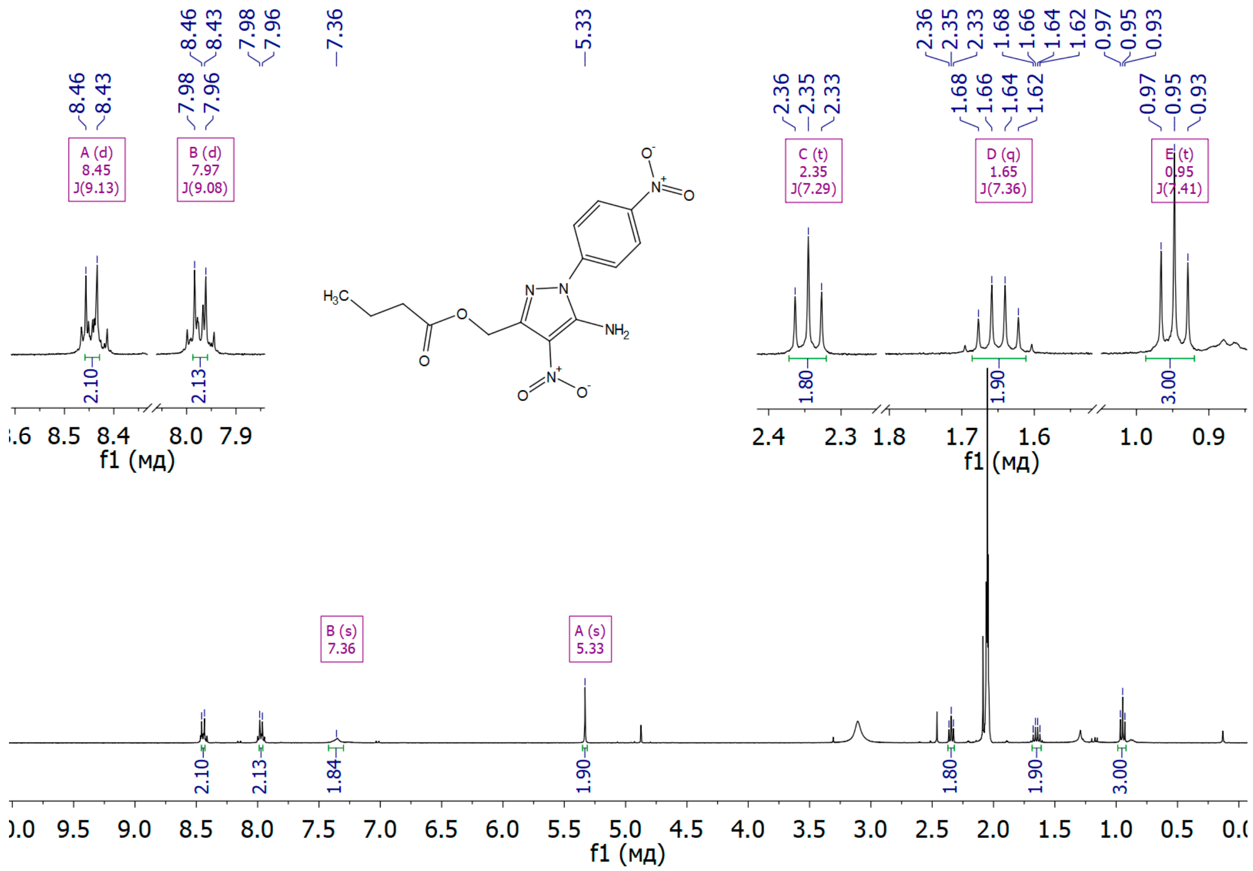

Figure S9.^{1}H NMR spectrum of **5c**(Acetone-d_6, 600 MHz)

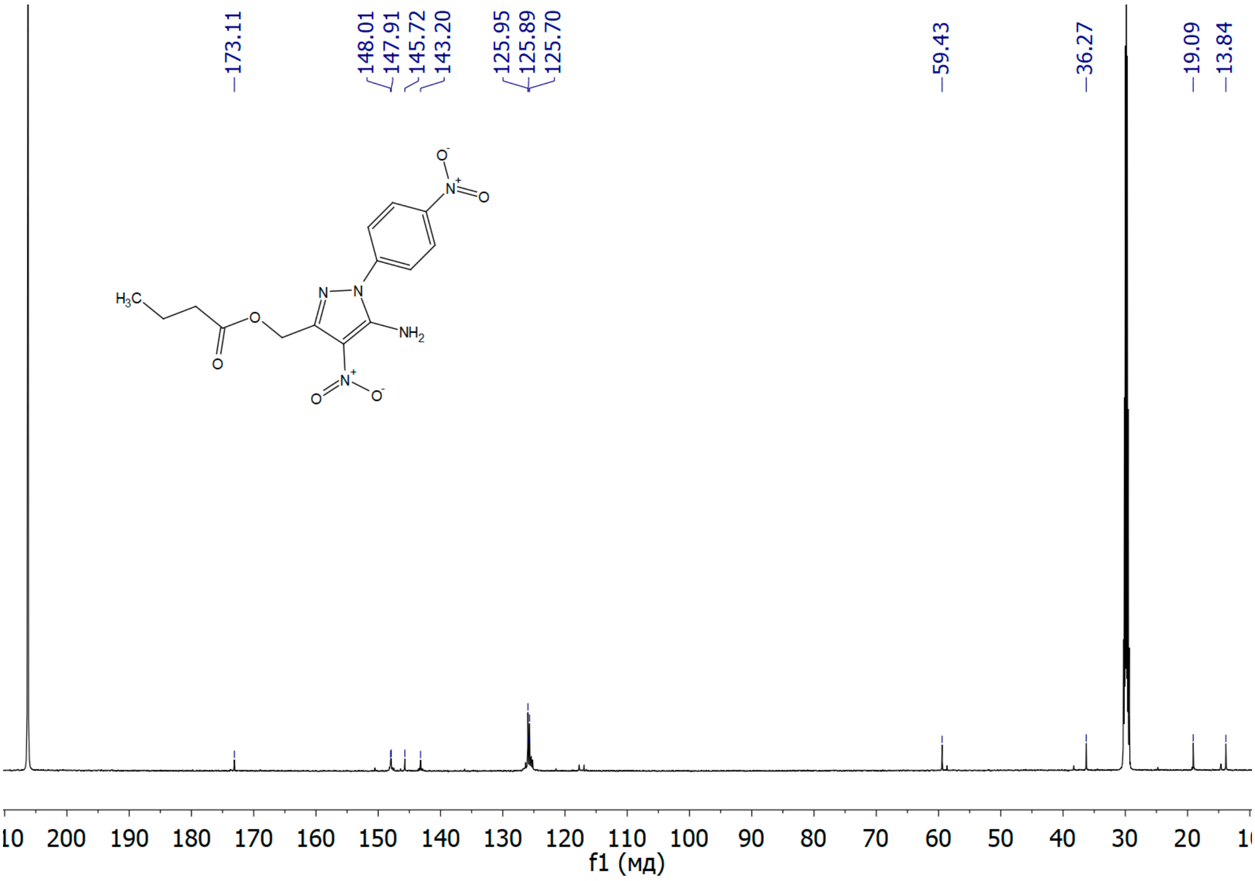

Figure S10.^{13}C NMR spectrum of **5c** (Acetone-d_6, 126 MHz)

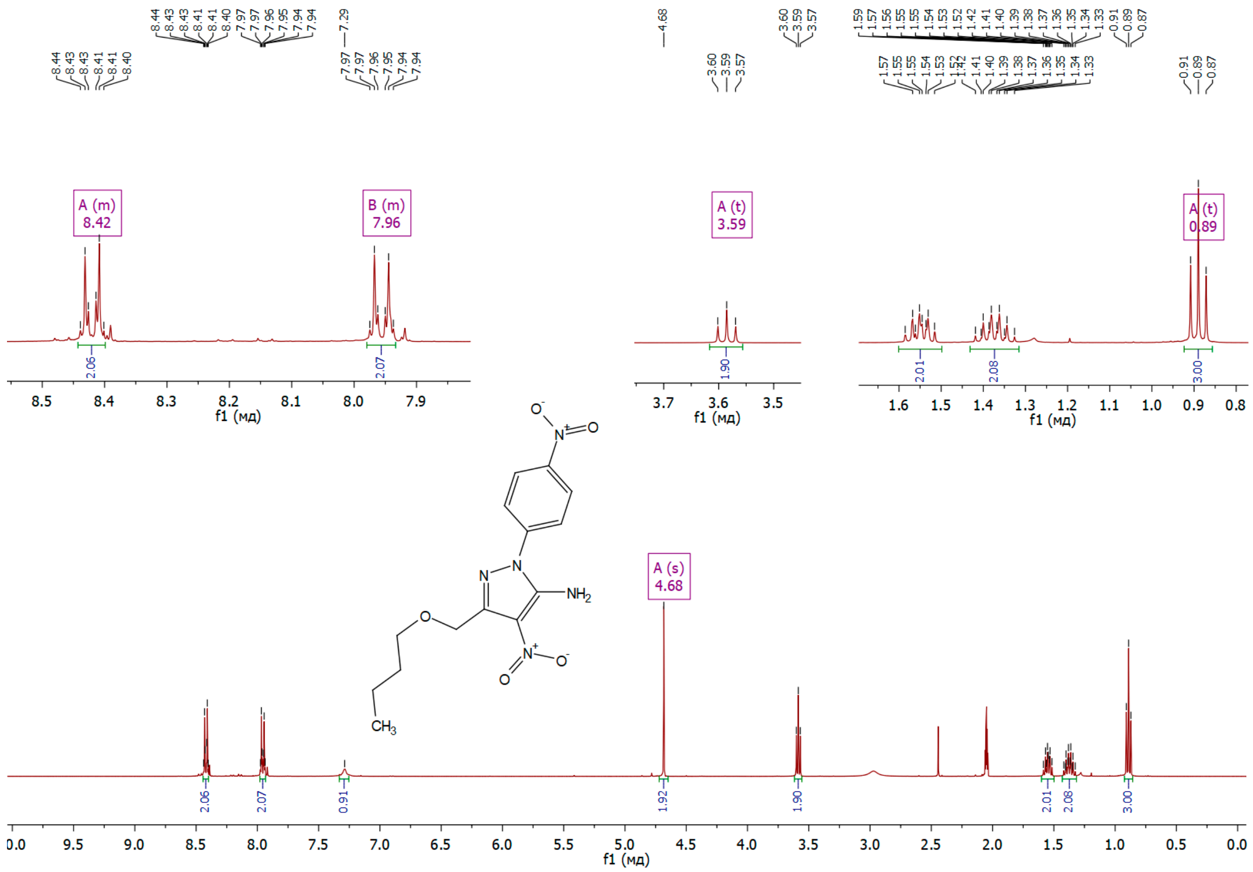

Figure S11.^{1}H NMR spectrum of **5d** (Acetone-*d*6, 400 MHz)

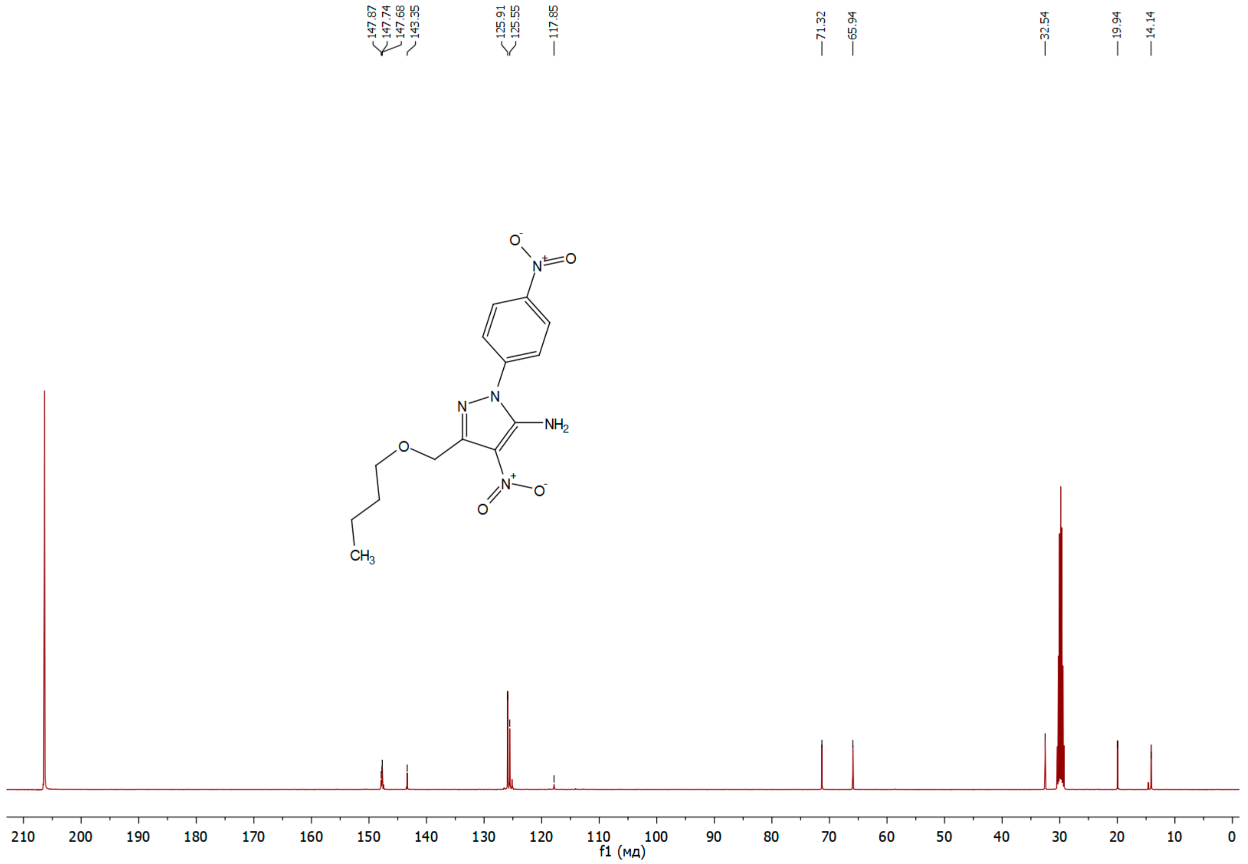

Figure S12.^{13}C NMR spectrum of **5d** (Acetone-*d*6, 101 MHz)

The X-ray diffraction data

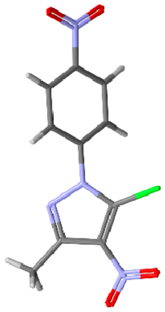

Figure S13. Molecular structure of compound **3**

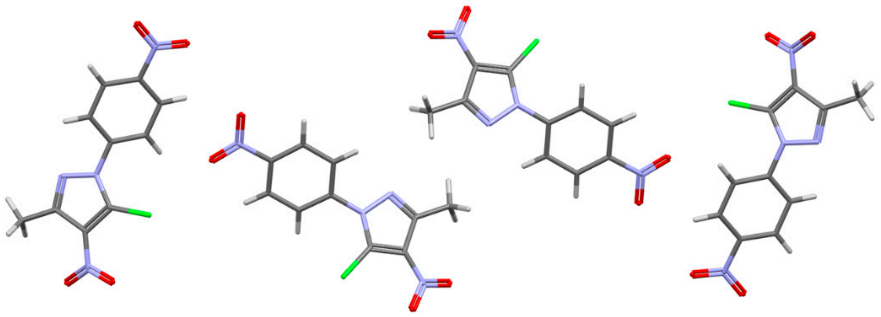

Figure S14. Unit cell of compound **3**(view along *a* axis)

Computational details

Computational Methods. Initial transition state calculations were performed in autodE v. 1.3.5.[1] Low energy conformers were located with the ETKDGv3 algorithm[2] implemented in RDKit v. 2022.09.5 and optimized using XTB then with Gaussian16 (G16, Revision C.01). [3]Single point energies were performed at the PBEO/D3BJ level in combination with the def2-TZVP[4] basis set. All structures were fully optimized with G16 at the (U)M06-2X/ 6-31+G(d,p)/UF level of theory. The implicit SMD solvation model was used to simulate acetic acid. Harmonic frequencies were used to verify geometry minima (no imaginary frequency), or saddle points (one imaginary frequency). Three-dimensionalstructureswereproducedwith CYLView.[5]

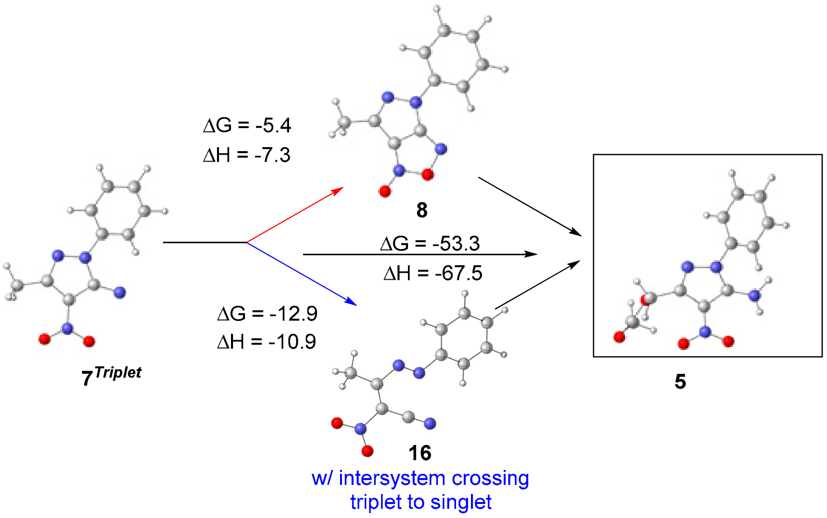

Scheme S1. The mechanistic study of the two possible pathways to lead to the same product **5**. From pyrazole nitrene to intermediate **16**, proceeds with intersystem crossing triplet to singlet.

There are two possible pathways that led to the observed product **5** from pyrazole nitrene.However, intermediate **16** can lead to formation of the byproduct **6**. The following Schemes S2 and S3 outline the full proposed mechanism from intermediates **8** and **16**.

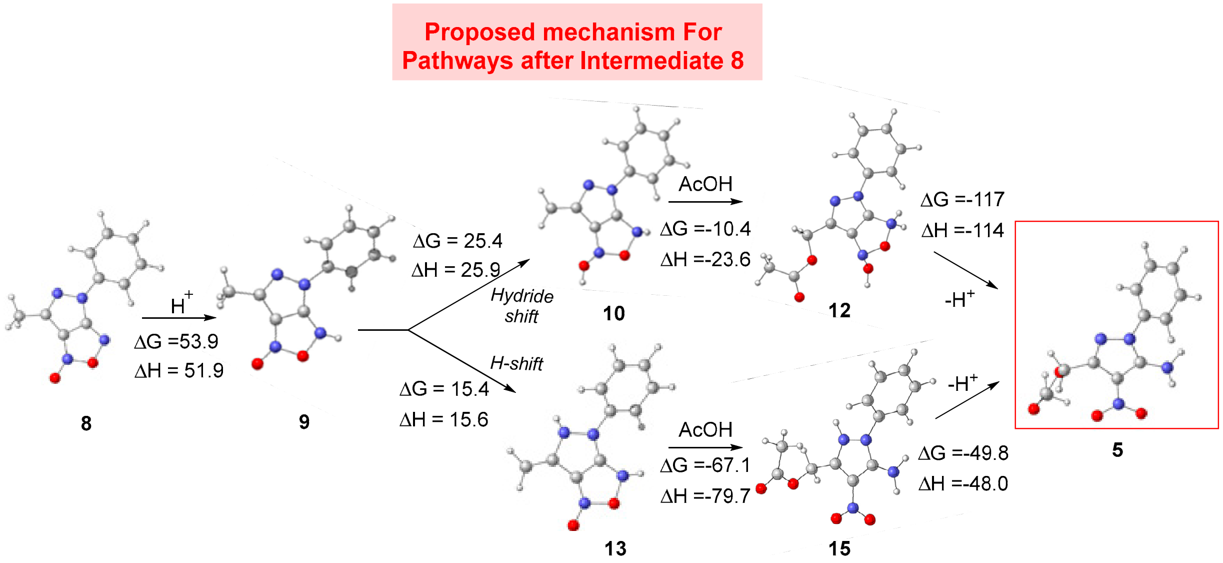

Scheme S2. Pathways from intermediate **8**.

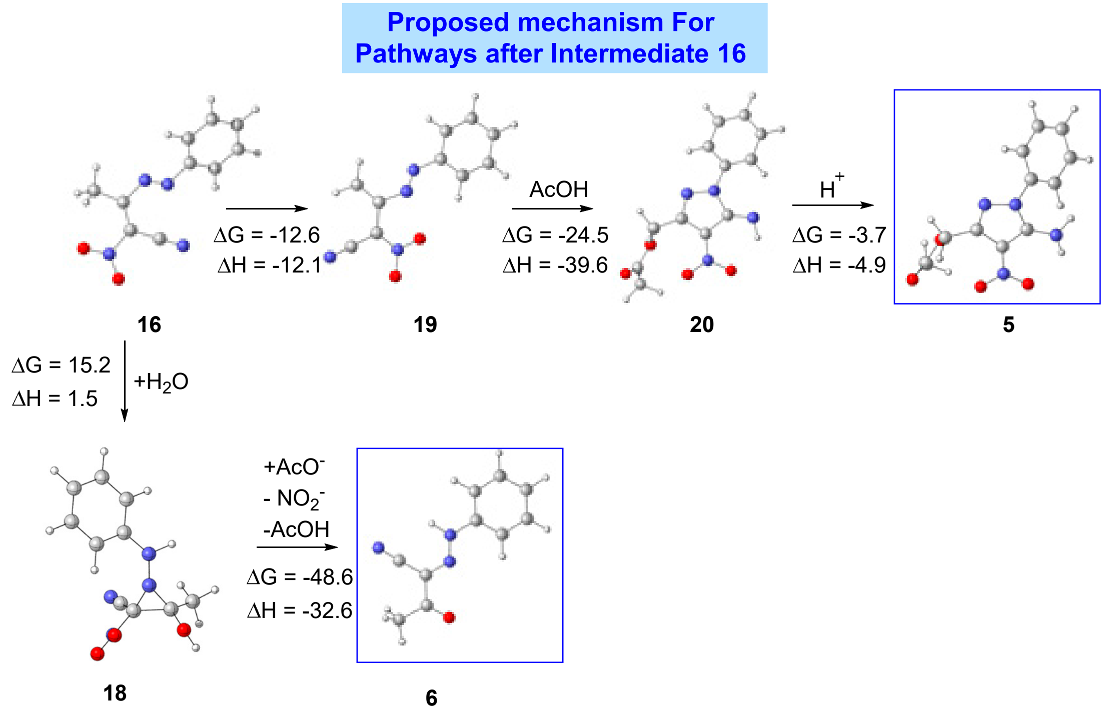

Scheme S3. Pathways from intermediate **16**.

XYZ Coordinates

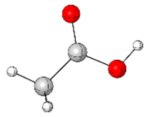

Acetic acid

Charge = 0

Multiplicity =1

Imaginary Frequency= 0

Zero-point Energies=-228.944951

E=-228.940403

H=-228.939459

G= -228.9720468

symmetry cs

C	0.000000000	0.145939000	0.000000000
C	-1.053899000	-0.914374000	0.000000000
O	-0.198918000	1.343956000	0.000000000
O	1.241110000	-0.368992000	0.000000000
H	-2.040494000	-0.453759000	0.000000000
H	-0.930515000	-1.546984000	0.883285000
H	-0.930515000	-1.546984000	-0.883285000
H	1.887383000	0.358623000	0.000000000

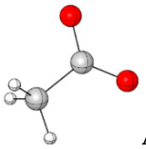

Acetic acid ion (-)

Charge = -1
Multiplicity =1
Imaginary Frequency= 0
Zero-point Energies=-228.480458
E= -228.476949
H=-228.476005
G=-228.506304
symmetry cs

C	0.000000000	0.181713000	0.000000000
C	-0.045154000	-1.345334000	0.000000000
O	-1.097340000	0.803276000	0.000000000
O	1.145255000	0.715251000	0.000000000
H	-1.072043000	-1.715833000	0.000000000
H	0.479824000	-1.725330000	0.881841000
H	0.479824000	-1.725330000	-0.881841000

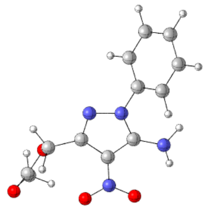

5 – Final Product

Charge = 0
Multiplicity =1
Imaginary Frequency= 0
Zero-point Energies=-983.736945
E=-983.719150
H=-983.718205
G=-983.784259
symmetry c1

N	1.301927000	2.268498000	0.496110000
C	0.696506000	1.139772000	0.099635000
C	-0.662886000	0.875089000	-0.150509000
N	-1.699661000	1.790365000	-0.006979000
O	-2.848078000	1.440791000	-0.279683000
O	-1.421185000	2.929863000	0.391007000
C	-0.736729000	-0.491533000	-0.543258000
C	-1.943352000	-1.287483000	-0.908731000
O	-2.782934000	-1.349155000	0.253118000
C	-4.101740000	-1.135113000	0.083705000
C	-4.806500000	-1.014219000	1.397970000
O	-4.622971000	-1.039977000	-1.006766000
N	0.457215000	-1.026032000	-0.541867000
N	1.327459000	-0.026281000	-0.170130000
C	2.719709000	-0.305914000	-0.048770000
C	3.120392000	-1.437889000	0.659120000
C	4.479404000	-1.718952000	0.768442000

C	5.423262000	-0.869146000	0.189994000
C	5.007311000	0.259194000	-0.515149000
C	3.649108000	0.542590000	-0.648723000
H	0.701703000	3.018204000	0.816454000
H	-1.645510000	-2.301307000	-1.184773000
H	-2.487600000	-0.822076000	-1.732516000
H	-4.449836000	-1.773978000	2.096053000
H	-5.881749000	-1.100198000	1.246997000
H	-4.573665000	-0.029097000	1.815665000
H	2.373159000	-2.083399000	1.109490000
H	4.800743000	-2.600603000	1.314025000
H	6.481889000	-1.090209000	0.284169000
H	5.737759000	0.914604000	-0.978863000
H	3.315072000	1.398428000	-1.228215000
H	2.253888000	2.233232000	0.837807000

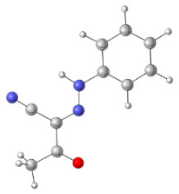

6 – Byproduct

Charge = 0

Multiplicity =1

Imaginary Frequency= 0

Zero-point Energies=-625.514691

E=-625.501904

H=-625.500960

G=-625.555762

symmetry c1

N	-0.290885000	0.611125000	0.008579000
N	0.662910000	-0.259384000	0.000469000
C	-1.646447000	0.220000000	0.004560000
C	1.916710000	0.108043000	0.003383000
C	2.962213000	-0.949047000	-0.013944000
C	4.389512000	-0.484662000	-0.015672000
O	2.649684000	-2.128252000	-0.026795000
C	-2.609766000	1.229391000	-0.032666000
C	-3.958257000	0.884176000	-0.036582000
C	-4.342659000	-0.455763000	-0.004642000
C	-3.366308000	-1.452615000	0.031863000
C	-2.013448000	-1.126517000	0.037223000
C	2.287824000	1.494090000	0.019696000
N	2.523870000	2.629610000	0.033929000
H	-0.086344000	1.613735000	0.013182000
H	4.588012000	0.124762000	0.872856000
H	5.052514000	-1.349622000	-0.027652000
H	4.579581000	0.142719000	-0.893553000
H	-2.305607000	2.272643000	-0.061274000
H	-4.708334000	1.668399000	-0.066046000
H	-5.394741000	-0.722088000	-0.007872000
H	-3.657366000	-2.498228000	0.057455000
H	-1.252700000	-1.898337000	0.067110000

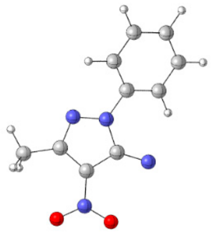

7 - Pyrazole nitrene Triplet

Charge = 0
Multiplicity =3
Imaginary Frequency= 0
Zero-point Energies= -754.685293
E=-754.672131
H=-754.671187
G=-754.727307
symmetry c1

C	-1.732623000	0.094974000	0.036378000
C	-2.486355000	1.137107000	-0.503405000
C	-3.871956000	1.017733000	-0.530580000
C	-4.491544000	-0.129354000	-0.033246000
C	-3.721860000	-1.159953000	0.504409000
C	-2.334211000	-1.052985000	0.551000000
N	-0.314758000	0.224872000	0.066223000
N	0.256595000	1.420483000	0.213217000
C	1.588341000	1.247724000	0.172040000
C	1.865710000	-0.107154000	-0.011770000
C	0.618392000	-0.791112000	-0.095694000
C	2.512015000	2.402605000	0.321944000
N	3.127206000	-0.733279000	-0.113547000
O	3.152934000	-1.947739000	-0.277814000
O	4.126565000	-0.027083000	-0.033039000
N	0.332470000	-2.049437000	-0.316260000
H	-1.989154000	2.015801000	-0.899318000
H	-4.467365000	1.821566000	-0.951575000
H	-5.572923000	-0.218143000	-0.061771000
H	-4.199525000	-2.048416000	0.904494000
H	-1.737997000	-1.839052000	1.003075000
H	1.928794000	3.315777000	0.448326000
H	3.153041000	2.494773000	-0.558897000
H	3.163105000	2.260290000	1.188610000

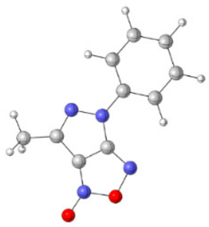

8 – Bicyclic Intermediate ($C_{10}N_4H_8O_2$)

Charge = 0

Multiplicity =1

Imaginary Frequency= 0

Zero-point Energies=-754.696140

E= -754.683765

H= -754.682821

G= -754.735967

symmetry c1

C	-3.199404000	-1.263677000	-1.540607000
C	-2.162699000	-0.538087000	-0.763299000
C	-2.325631000	0.555310000	0.162520000
C	-1.038228000	0.882497000	0.631965000
N	-1.038857000	1.873703000	1.489444000
O	-2.382752000	2.205639000	1.581042000
N	-3.157238000	1.384920000	0.758798000
O	-4.370971000	1.574028000	0.746084000
N	-0.887197000	-0.812850000	-0.819748000
N	-0.175392000	0.029346000	0.014467000
C	1.229286000	-0.056755000	0.129061000
C	1.886763000	0.773424000	1.039227000
C	3.272989000	0.693619000	1.149246000
C	3.997460000	-0.203457000	0.366663000
C	3.323267000	-1.027173000	-0.534822000
C	1.938806000	-0.962093000	-0.662062000
H	-3.735977000	-0.570397000	-2.194603000
H	-3.927775000	-1.721108000	-0.865057000
H	-2.729063000	-2.040402000	-2.145043000
H	1.331471000	1.473211000	1.655749000
H	3.783660000	1.339940000	1.856371000
H	5.077351000	-0.260519000	0.458075000
H	3.876732000	-1.730021000	-1.150079000
H	1.413498000	-1.599299000	-1.363293000

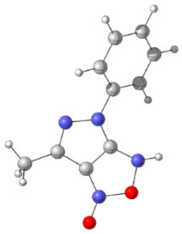

9 – Intermediate ion (+)

Zero-point Energies= -755.076982
E= -755.064470
H= -755.063526
G= -755.116161
Charge = 1
Multiplicity =1
Imaginary Frequency= 0
symmetry c1

C	3.159455000	1.995846000	1.002161000
C	2.131331000	0.993331000	0.635918000
C	2.307939000	-0.354066000	0.149287000
C	1.026269000	-0.875494000	-0.035864000
N	1.098478000	-2.150651000	-0.474141000
O	2.455244000	-2.393073000	-0.712683000
N	3.178081000	-1.264320000	-0.289806000
O	4.366225000	-1.329671000	-0.367301000
N	0.845138000	1.201529000	0.721491000
N	0.156147000	0.056201000	0.312269000
C	-1.271886000	0.028893000	0.289336000
C	-1.924399000	-1.180343000	0.515697000
C	-3.315974000	-1.205000000	0.470725000
C	-4.030750000	-0.034505000	0.220615000
C	-3.354777000	1.167761000	0.011418000
C	-1.963611000	1.210383000	0.039137000
H	2.672147000	2.916017000	1.325418000
H	3.802244000	2.204573000	0.142429000
H	3.786212000	1.609935000	1.810788000
H	0.530574000	-2.534943000	-1.237025000
H	-1.365246000	-2.081603000	0.753138000
H	-3.838382000	-2.139239000	0.648713000
H	-5.115502000	-0.057769000	0.194517000
H	-3.910380000	2.079763000	-0.181171000
H	-1.424578000	2.136344000	-0.128465000

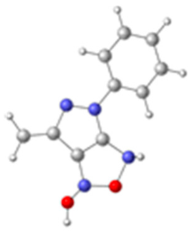

10 – intermediate ion (+)

Charge = 1
Multiplicity =1
Imaginary Frequency= 0
Zero-point Energies=-755.035747
E= -755.023233
H= -755.022289
G= -755.075621
symmetry c1

C	-2.295423000	2.776413000	-0.080972000
C	-1.578399000	1.632313000	0.028217000
C	-1.938754000	0.260405000	0.229880000
C	-0.791853000	-0.431532000	0.235316000
N	-0.980710000	-1.808585000	0.297496000
O	-2.406027000	-1.831583000	0.682688000
N	-3.046682000	-0.627823000	0.285049000
O	-3.465451000	-0.773402000	-1.048554000
N	-0.186100000	1.683442000	-0.067807000
N	0.248260000	0.474640000	0.036423000
C	1.643214000	0.166399000	-0.013268000
C	2.566026000	1.181430000	0.238910000
C	3.917396000	0.868534000	0.172644000
C	4.327303000	-0.429778000	-0.140330000
C	3.385086000	-1.426550000	-0.390171000
C	2.025944000	-1.136865000	-0.326962000
H	-3.379698000	2.757262000	-0.026009000
H	-1.781776000	3.722505000	-0.227153000
H	-0.538461000	-2.246264000	1.112946000
H	-4.400027000	-1.031306000	-0.983285000
H	2.230447000	2.181202000	0.489951000
H	4.652907000	1.640555000	0.371241000
H	5.385897000	-0.663869000	-0.188978000
H	3.703226000	-2.432210000	-0.642855000
H	1.292697000	-1.904333000	-0.556649000

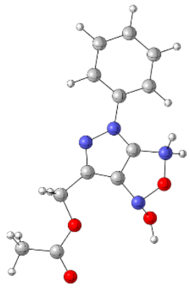

12 – Intermediate ion (+)

Charge = 1

Multiplicity =1

Imaginary Frequency= 0

Zero-point Energies=-984.017563

E=-984.000225

H=-983.999281

G=-984.064255

symmetry c1

C	-0.827485000	-0.674562000	-0.098112000
C	-0.694948000	0.712151000	-0.279674000
C	0.636848000	0.943983000	-0.211869000
O	-0.395326000	2.834402000	-0.833919000
N	-1.433068000	1.927053000	-0.417993000
O	-1.822442000	2.300863000	0.857106000
N	0.379949000	-1.210054000	0.052003000
N	1.289457000	-0.211251000	-0.016859000
C	2.691764000	-0.469625000	0.062411000
C	3.182032000	-1.667918000	-0.449982000
C	4.550506000	-1.911022000	-0.379222000
C	5.409801000	-0.964720000	0.180202000
C	4.898676000	0.228240000	0.687855000
C	3.529082000	0.479625000	0.642270000
H	-2.650496000	2.796860000	0.733914000
H	2.502833000	-2.386789000	-0.894783000
H	4.946015000	-2.841592000	-0.773057000
H	6.476222000	-1.160475000	0.226132000
H	5.560757000	0.959505000	1.139786000
H	3.127347000	1.384934000	1.089109000
C	-2.080844000	-1.485908000	-0.069246000
H	-2.049122000	-2.200431000	0.758101000
H	-2.209438000	-2.020415000	-1.015714000
O	-3.124220000	-0.527219000	0.111135000
C	-4.421927000	-0.904062000	0.141621000
O	-5.246590000	-0.027675000	0.277549000
C	-4.744776000	-2.360996000	0.009373000
H	-4.400558000	-2.742169000	-0.956783000
H	-4.252596000	-2.937161000	0.798086000
H	-5.823937000	-2.482520000	0.085043000
N	0.904915000	2.350544000	-0.369769000
H	1.165192000	2.841826000	0.502988000
H	1.555268000	2.600303000	-1.133215000

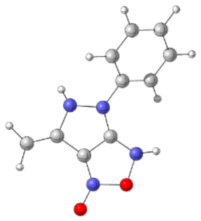

13 – intermediate ion (+)

Charge = 1
Multiplicity =1
Imaginary Frequency= 0
Zero-point Energies=-755.052196
E=-755.039688
H=-755.038743
G=-755.091591
symmetry c1

C	2.578090000	2.599780000	0.221118000
C	1.722531000	1.579792000	0.176512000
C	1.964572000	0.152772000	0.099256000
C	0.723704000	-0.505824000	0.087973000
N	0.905146000	-1.833113000	-0.044088000
O	2.282417000	-2.023887000	-0.249025000
N	2.900954000	-0.729491000	-0.152491000
O	4.100981000	-0.702413000	-0.281274000
N	0.279945000	1.681983000	0.171943000
N	-0.257038000	0.358157000	0.168076000
C	-1.660753000	0.147199000	0.072294000
C	-2.185034000	-1.057254000	0.540506000
C	-3.554836000	-1.274019000	0.425588000
C	-4.379122000	-0.294539000	-0.128111000
C	-3.833799000	0.910359000	-0.570352000
C	-2.464539000	1.144245000	-0.476557000
H	3.643792000	2.402992000	0.231117000
H	2.224066000	3.623960000	0.254641000
H	0.360982000	-2.418232000	-0.686674000
H	-1.550952000	-1.800947000	1.014955000
H	-3.977432000	-2.206081000	0.785898000
H	-5.447192000	-0.468361000	-0.208729000
H	-4.473904000	1.674508000	-0.999002000
H	-2.027615000	2.070220000	-0.833461000
H	-0.086861000	2.174535000	0.990206000

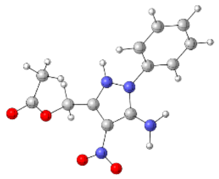

15 – Intermediate ion (+)

Charge = 1
Multiplicity =1
Imaginary Frequency= 0
Zero-point Energies=-984.123698
E= -984.106145
H=-984.105201
G=-984.170620
symmetry c1

N	0.290765000	0.618360000	0.006193000
N	-0.661404000	-0.251608000	0.078220000
C	1.646455000	0.224816000	-0.001264000
C	-1.915769000	0.112183000	0.045088000
C	-2.960260000	-0.942860000	0.131492000
C	-4.385473000	-0.495693000	-0.018997000
O	-2.649469000	-2.107778000	0.318095000
C	2.610102000	1.222944000	0.149325000
C	3.957904000	0.875494000	0.142049000
C	4.340514000	-0.457137000	-0.007417000
C	3.363968000	-1.443143000	-0.157191000
C	2.011970000	-1.112703000	-0.161499000
C	-2.288689000	1.492740000	-0.073881000
N	-2.527911000	2.630633000	-0.170094000
H	0.084981000	1.616168000	-0.088309000
H	-4.512340000	0.081628000	-0.940655000
H	-5.039365000	-1.367647000	-0.029454000
H	-4.656670000	0.157246000	0.818751000
H	2.304600000	2.258755000	0.272499000
H	4.708592000	1.650925000	0.258741000
H	5.391997000	-0.725898000	-0.009754000
H	3.654522000	-2.481999000	-0.279928000
H	1.250299000	-1.874333000	-0.285177000

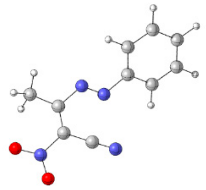

16 - Intermediate

Charge = 0

Multiplicity =1

Imaginary Frequency= 0

Zero-point Energies= -754.703640

E= -754.689467

G= -754.688523

H= -754.747241

symmetry c1

C	-2.020548000	0.126608000	0.241756000
C	-2.641478000	0.875860000	-0.766815000
C	-4.006322000	0.727139000	-0.966339000
C	-4.744451000	-0.157750000	-0.170688000
C	-4.119457000	-0.898245000	0.830668000
C	-2.750339000	-0.755075000	1.041234000
N	-0.632943000	0.178992000	0.530635000
N	0.036299000	0.954497000	-0.173785000
C	1.421851000	0.962594000	0.061096000
C	2.093878000	-0.209744000	-0.091201000
C	1.459481000	-1.448781000	-0.419552000
C	1.978655000	2.314575000	0.349011000
N	3.539461000	-0.302576000	0.006080000
O	4.031335000	-1.413406000	-0.111898000
O	4.178541000	0.717518000	0.201310000
N	0.946580000	-2.443951000	-0.710554000
H	-2.057798000	1.558151000	-1.375697000
H	-4.503140000	1.299286000	-1.743492000
H	-5.812029000	-0.266423000	-0.336968000
H	-4.694161000	-1.584124000	1.444415000
H	-2.232168000	-1.319118000	1.811079000
H	1.153946000	3.021635000	0.444418000
H	2.649916000	2.636298000	-0.452738000
H	2.563030000	2.289573000	1.272043000

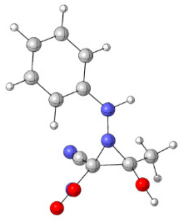

18 - Intermediate

Charge = 0
Multiplicity =1
Imaginary Frequency= 0
Zero-point Energies=-831.081693
E=-831.066448
H=-831.065504
G=-831.124547
symmetry c1

N	0.497811000	1.203618000	-0.141750000
N	-0.572139000	0.458063000	-0.702078000
C	1.740180000	0.521791000	-0.182475000
C	-1.880832000	1.019686000	-0.554513000
C	-1.425318000	-0.173365000	0.294933000
C	-2.127209000	2.341013000	0.118416000
O	-2.660432000	0.736856000	-1.647715000
C	2.897968000	1.272020000	0.057974000
C	4.135500000	0.641672000	0.094218000
C	4.234573000	-0.739218000	-0.096183000
C	3.078904000	-1.477761000	-0.331267000
C	1.828665000	-0.857666000	-0.378340000
N	-1.889172000	-1.537907000	-0.053218000
O	-1.694888000	-1.938946000	-1.177590000
O	-2.481419000	-2.139052000	0.827202000
C	-1.185838000	-0.005191000	1.706064000
N	-0.976587000	0.160999000	2.829761000
H	0.537099000	2.106579000	-0.611696000
H	-1.956462000	3.135238000	-0.614175000
H	-3.167497000	2.385216000	0.450897000
H	-1.473179000	2.493492000	0.977419000
H	-3.576077000	1.007843000	-1.479221000
H	2.818899000	2.344166000	0.219127000
H	5.028272000	1.232540000	0.276583000
H	5.202262000	-1.229368000	-0.062495000
H	3.139679000	-2.550836000	-0.487033000
H	0.941957000	-1.447034000	-0.586539000

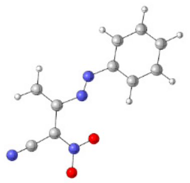

19 – Intermediate ion (-)

Charge = -1

Multiplicity =1

Imaginary Frequency= 0

Zero-point Energies= -754.258985

E= -754.245328

G= -754.244384

H= -754.301627

symmetry c1

N	0.824295000	-0.886408000	0.436185000
N	-0.101837000	-0.428088000	-0.248210000
C	2.107105000	-0.342231000	0.118610000
C	-1.386336000	-0.995304000	0.040130000
C	-1.535358000	-2.322396000	0.166840000
C	-2.501575000	-0.055154000	-0.040984000
N	-2.396641000	1.224538000	0.405862000
C	-3.763546000	-0.457902000	-0.526306000
O	-3.366005000	2.016247000	0.272027000
O	-1.332621000	1.609421000	0.951027000
N	-4.785499000	-0.841119000	-0.929752000
C	2.274653000	0.911377000	-0.478806000
C	3.561681000	1.375919000	-0.728353000
C	4.672378000	0.597757000	-0.389944000
C	4.496337000	-0.644894000	0.218115000
C	3.211142000	-1.110507000	0.488090000
H	-0.678731000	-2.981282000	0.078667000
H	-2.517453000	-2.762325000	0.307899000
H	1.401751000	1.510268000	-0.718256000
H	3.701510000	2.352474000	-1.182249000
H	5.673838000	0.967684000	-0.589096000
H	5.358004000	-1.246509000	0.490968000
H	3.048971000	-2.068113000	0.974678000

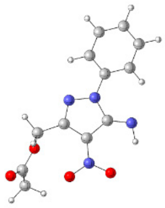

20 – Intermediate ion (-)

Charge = -1

Multiplicity =1

Imaginary Frequency= 0

Zero-point Energies=-983.265217

E=-983.247947

H= -983.247003

G= -983.312672

symmetry c1

N	-1.312489000	-2.470978000	0.380485000
C	-0.635667000	-1.439070000	0.000112000
C	0.756769000	-1.237943000	-0.358605000
N	1.709006000	-2.201453000	-0.400865000
O	2.877844000	-1.914618000	-0.758902000
O	1.409425000	-3.378554000	-0.080507000
C	0.913087000	0.148968000	-0.665795000
C	2.144828000	0.875779000	-1.085308000
O	3.078509000	0.803639000	0.005670000
C	4.356311000	0.510383000	-0.280489000
C	5.136884000	0.202778000	0.959318000
O	4.800737000	0.490950000	-1.410371000
N	-0.216031000	0.780007000	-0.529577000
N	-1.166680000	-0.148155000	-0.153384000
C	-2.469947000	0.307034000	0.122697000
C	-2.679132000	1.673878000	0.360934000
C	-3.962063000	2.146544000	0.617174000
C	-5.051139000	1.274814000	0.647392000
C	-4.836961000	-0.081265000	0.409675000
C	-3.560027000	-0.573296000	0.143865000
H	-0.668047000	-3.258854000	0.421539000
H	1.908258000	1.925436000	-1.274987000
H	2.589052000	0.425750000	-1.974411000
H	4.878602000	0.899254000	1.759306000
H	6.203747000	0.238428000	0.742013000
H	4.862155000	-0.806726000	1.282850000
H	-1.836066000	2.353706000	0.343790000
H	-4.106646000	3.207393000	0.801241000
H	-6.049931000	1.647778000	0.852531000
H	-5.672895000	-0.774995000	0.423508000
H	-3.401593000	-1.626513000	-0.040499000

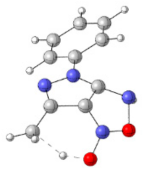

9 →10 Transition State

Charge = 0

Multiplicity =1

Imaginary Frequency= 1 (-1312.0817)

Zero-point Energies= -754.703640

E= -754.689467

H= -754.688523

G= -754.747241

symmetry c1

C	2.778990000	2.136566000	-0.052553000
C	1.706526000	1.353440000	-0.486523000
C	1.885215000	-0.084263000	-0.649572000
C	0.622456000	-0.671832000	-0.523928000
N	0.771602000	-1.958670000	-0.002568000
O	2.125891000	-1.907422000	0.619794000
N	2.715590000	-0.770881000	0.097188000
O	3.698583000	-0.228227000	0.703669000
N	0.378828000	1.571344000	-0.430801000
N	-0.226961000	0.347158000	-0.392653000
C	-1.624608000	0.223661000	-0.175258000
C	-2.311350000	1.261372000	0.456460000
C	-3.667445000	1.089759000	0.704189000
C	-4.311334000	-0.094020000	0.334725000
C	-3.606835000	-1.115746000	-0.302825000
C	-2.252152000	-0.959648000	-0.574824000
H	3.385910000	0.890148000	0.769340000
H	2.567940000	3.074540000	0.449784000
H	3.731527000	2.058138000	-0.575637000
H	0.135895000	-2.187853000	0.769196000
H	-1.785886000	2.164245000	0.744685000
H	-4.223898000	1.880648000	1.194582000
H	-5.370489000	-0.215370000	0.534618000
H	-4.115731000	-2.021551000	-0.612700000
H	-1.711254000	-1.716147000	-1.139083000

References

(1) Young, T. A.; Silcock, J. J.; Sterling, A. J.; Duarte, F. autodE: Automated Calculation of Reaction Energy Profiles— Application to Organic and Organometallic Reactions. *Angew. Chem. Int. Ed.***2021**, *60* (8), 4266–4274. https://doi.org/10.1002/anie.202011941.

(2) Riniker, S.; Landrum, G. A. Better Informed Distance Geometry: Using What We Know To Improve Conformation Generation. *J. Chem. Inf. Model.***2015**, *55* (12), 2562–2574. https://doi.org/10.1021/acs.jcim.5b00654.

(3) Gaussian 16, Revision C.01, Frisch, M. J.; Trucks, G. W.; Schlegel, H. B.; Scuseria, G. E.; Robb, M. A.; Cheeseman, J. R.; Scalmani, G.; Barone, V.; Petersson, G. A.; Nakatsuji, H.; Li, X.; Caricato, M.; Marenich, A. V.; Bloino, J.; Janesko, B. G.; Gomperts, R.; Mennucci, B.; Hratchian, H. P.; Ortiz, J. V.; Izmaylov, A. F.; Sonnenberg, J. L.; Williams-Young, D.; Ding, F.; Lipparini, F.; Egidi, F.; Goings, J.; Peng, B.; Petrone, A.; Henderson, T.; Ranasinghe, D.; Zakrzewski, V. G.; Gao, J.; Rega, N.; Zheng, G.; Liang, W.; Hada, M.; Ehara, M.; Toyota, K.; Fukuda, R.; Hasegawa, J.; Ishida, M.; Nakajima, T.; Honda, Y.; Kitao, O.; Nakai, H.; Vreven, T.; Throssell, K.; Montgomery, J. A., Jr.; Peralta, J. E.; Ogliaro, F.; Bearpark, M. J.; Heyd, J. J.; Brothers, E. N.; Kudin, K. N.; Staroverov, V. N.; Keith, T. A.; Kobayashi, R.; Normand, J.; Raghavachari, K.; Rendell, A. P.; Burant, J. C.; Iyengar, S. S.; Tomasi, J.; Cossi, M.; Millam, J. M.; Klene, M.; Adamo, C.; Cammi, R.; Ochterski, J. W.; Martin, R. L.; Morokuma, K.; Farkas, O.; Foresman, J. B.; Fox, D. J. Gaussian, Inc., Wallingford CT, 2016. .

(4) Weigend, F.; Ahlrichs, R. Balanced Basis Sets of Split Valence, Triple Zeta Valence and Quadruple Zeta Valence Quality for H to Rn: Design and Assessment of Accuracy. *Phys. Chem. Chem. Phys.***2005**, *7* (18), 3297–3305. https://doi.org/10.1039/B508541A.

(5) *CYLview20; Legault, C. Y., Université de Sherbrooke, 2020 (http://www.cylview.org)*.
